# Inhibiting NLRP3 signaling in aging podocytes improves their life- and health-span

**DOI:** 10.18632/aging.204897

**Published:** 2023-07-23

**Authors:** Natalya Kaverina, R. Allen Schweickart, Gek Cher Chan, Joseph C. Maggiore, Diana G. Eng, Yuting Zeng, Sierra R. McKinzie, Hannah S. Perry, Adilijiang Ali, Christopher O’Connor, Beatriz Maria Veloso Pereira, Ashleigh B. Theberge, Joshua C. Vaughan, Carol J. Loretz, Anthony Chang, Neil A. Hukriede, Markus Bitzer, Jeffrey W. Pippin, Oliver Wessely, Stuart J. Shankland

**Affiliations:** 1 Division of Nephrology, University of Washington, Seattle, WA 98109, USA; 2 Lerner Research Institute, Cleveland Clinic Foundation, Cleveland, OH 44106, USA; 3 Department of Medicine, Division of Nephrology, National University Hospital, Singapore; 4 Department of Developmental Biology, University of Pittsburgh, Pittsburgh, PA 15261, USA; 5 Department of Chemistry, University of Washington, Seattle, WA 98109, USA; 6 Division of Nephrology, University of Michigan, Ann Arbor, MI 48109, USA; 7 Department of Physiology and Biophysics, University of Washington, Seattle, WA 98109, USA; 8 Department of Pathology, University of Chicago, Chicago, IL 60637, USA; 9 Institute for Stem Cell and Regenerative Medicine, University of Washington, Seattle, WA 98109, USA

**Keywords:** kidney, podocyte, NLRP3 inflammasome, aging, reporter

## Abstract

The decrease in the podocyte’s lifespan and health-span that typify healthy kidney aging cause a decrease in their normal structure, physiology and function. The ability to halt and even reverse these changes becomes clinically relevant when disease is superimposed on an aged kidney. RNA-sequencing of podocytes from middle-aged mice showed an inflammatory phenotype with increases in the NLRP3 inflammasome, signaling for IL2/Stat5, IL6 and TNF, interferon gamma response, allograft rejection and complement, consistent with inflammaging. Furthermore, injury-induced NLRP3 signaling in podocytes was further augmented in aged mice compared to young ones. The NLRP3 inflammasome (NLRP3, Caspase-1, IL1β IL-18) was also increased in podocytes of middle-aged humans. Higher transcript expression for NLRP3 in human glomeruli was accompanied by reduced podocyte density and increased global glomerulosclerosis and glomerular volume. Pharmacological inhibition of NLRP3 with MCC950, or gene deletion, reduced podocyte senescence and the genes typifying aging in middle-aged mice, which was accompanied by an improved podocyte lifespan and health-span. Moreover, modeling the injury-dependent increase in NLRP3 signaling in human kidney organoids confirmed the anti-senescence effect of MC9950. Finally, NLRP3 also impacted liver aging. Together, these results suggest a critical role for the NLRP3 inflammasome in podocyte and liver aging.

## INTRODUCTION

As the world’s population ages and life expectancy increases, the impact of aging on kidney health becomes a more critical concern. In fact, the incidence and prevalence of chronic and end-stage kidney disease is highest in the elderly [1–6]. Moreover, kidney diseases superimposed on aged kidneys have worse outcomes than the same disease in younger kidneys [7, 8]. Glomerular Filtration Rate (GFR) declines after age 40 by 0.8–1.0% per year [9–11], and kidneys from 70–75-year-old healthy individuals have 48% fewer intact nephrons compared to 19–29-year-old individuals, a number consistent with an estimated annual loss of 6,000–6,500 nephrons after age 30 [12–14]. Typical age-induced glomerulosclerosis is closely associated with a decrease in the lifespan and health-span of the post-mitotic glomerular epithelial cells called podocytes [15–17]. Podocyte aging involves canonical aging players such as p16 Ink4a, p53 and telomere shortening [15]. In addition, recent studies have identified critical roles for GSK3β [18] and programmed cell death protein-1 [19] as mechanisms contributing more specifically to podocyte aging. Yet, these do not explain the entirety of the podocyte damage in aged kidneys.

To gain insights into additional candidate mechanisms, we recently performed transcriptome analysis comparing podocytes from young mice (3-months-old, ~20-years-old in humans) to aged mice (24-months-old mice, ~70^+^-years-old in humans) [20]. Among the most dramatic changes was a statistically significant increase in transcripts for the Nod-like receptor protein 3 (NLRP3), a key protein of the inflammasome and its downstream effectors *Caspase-1* and *Interleukin-1β* [21]. Although the NLRP3 inflammasome was initially considered restricted to immune cells following induction by DAMPs and PAMPs [22], it has gained wider importance as being the cause of sterile inflammation in non-immune cells including podocytes [21]. NLRP3 expression is increased in podocytes and contributes to their damage in APOL-1 associated podocytopathy [23], diabetic kidney disease [24–29], lupus nephritis [30, 31], several primary nephrotic syndromes [32–34] and complement- [35] aldosterone- [36] and angiotensin II- [37] induced injury.

However, the functional consequence of increased levels of NLRP3 in aged podocytes is unknown. Based on the increased levels in podocytes of mice with advanced age, [20] we hypothesized that reducing NLRP3 signaling earlier at middle-age improves overall podocyte health and slows down healthy podocyte aging in mice. To this end, we performed a comprehensive analysis of inflammasome signaling including pharmacological and genetic NLRP3 loss-of-function approaches.

## RESULTS

### Middle-aged podocytes acquire an inflammatory phenotype

**Figure 1:**
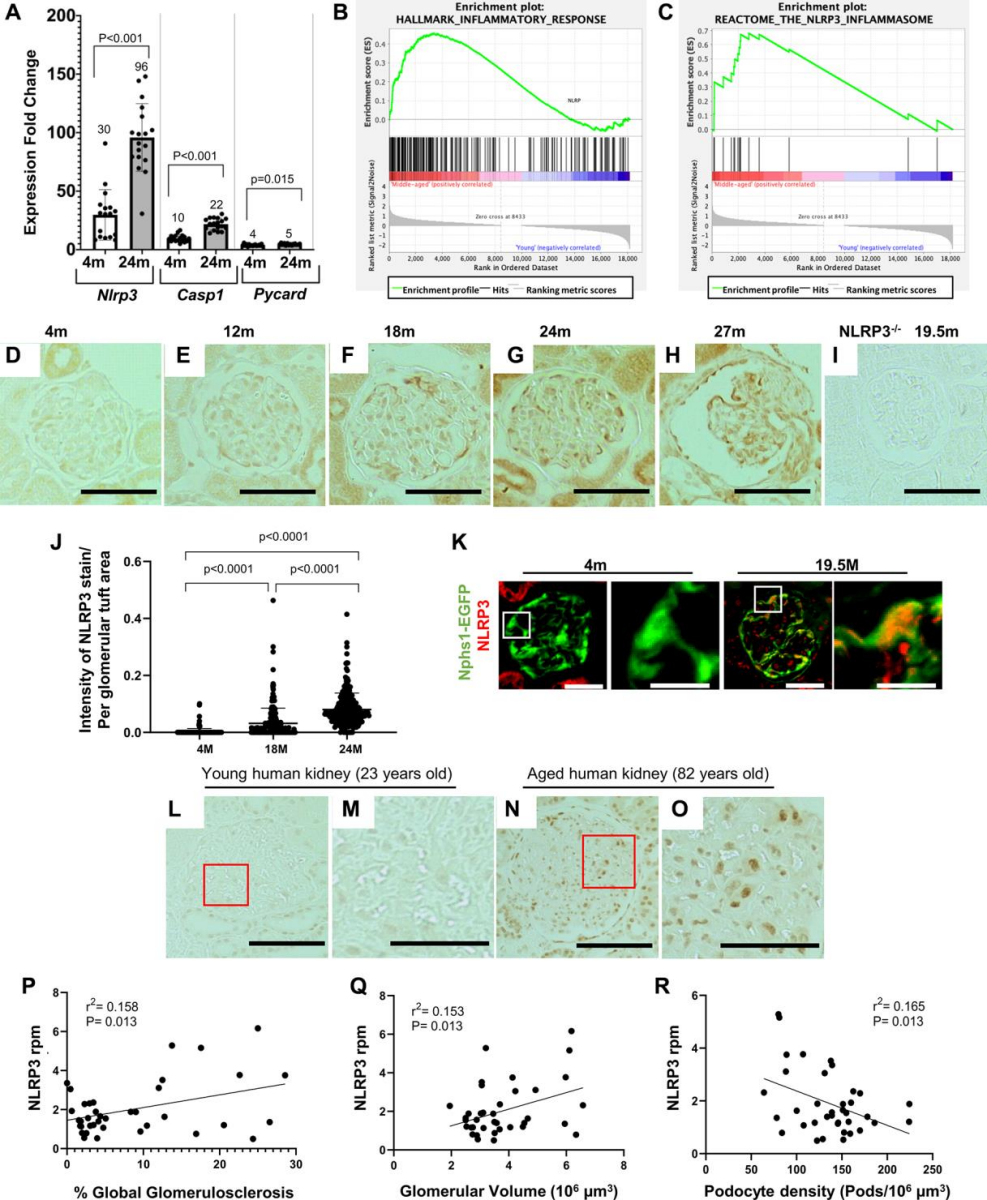
**Increase in the NLRP3 inflammasome in middle-aged podocytes.** (**A**) qRT-PCR of inflammasome components *Nlrp3*, *Casp1* and *Pycard* comparing podocytes from 4 months (m)-old to 24 m-old mice. Graph shows increase in the NLRP3 signaling. Error bars correspond to standard deviation; data are compared using Student’s test and *p*-values are indicated above the bars. (**B**, **C**) GSEA plots comparing podocytes isolated from middle-aged to young mice show enrichment of inflammatory response (**B**) and the NLRP3 inflammasome (**C**) gene sets. (**D**–**J**) Nlrp3 immunoperoxidase staining (brown) comparing glomeruli of differently aged mice with no staining at 4 m (**D**) and 12 m (**E**), but increasing staining at 18 m (**F**), 24 m (**G**) and 27 m (**H**). Quantification of the staining intensity is shown in (**J**). NLRP3 staining was absent in NLRP3 null (−/−) mice aged 19.5 m (**I**). (**K**) NLRP3 immunofluorescent staining (red) was absent in podocytes (labeled in green) of young, 4 m-old Nphs1-EGFP reporter mice, but colocalized to podocytes of 24 m-old aged mice (labeled in yellow/orange). (**L**–**O**) NLRP3 immunostaining of human kidneys shows no staining in young (23 years old) glomeruli (**L**, **M**), but is detected in aged (82 years old) glomeruli (**N**, **O**). Panels (**M**) and (**O**) show enlarged views indicated by the red boxes in (**L**) and (**N**), respectively. (**P**–**R**) NLRP3 transcripts from micro-dissected human glomeruli. Higher Expression of NLRP3 is associated with increased percent of globally sclerosed glomeruli (Figure 1P), higher glomerular volume (Figure 1Q) and reduced podocyte density (Figure 1R).

We have previously reported that aged 22 to 24-month-old (~70 human years) podocytes acquire an inflammatory phenotype including upregulation of NLRP3 signaling as measured by mRNA sequencing of isolated podocytes [20]. Based on these and published data on the importance of NLRP3 signaling, we further investigated the NLRP3 inflammasome in podocyte aging. First, the mRNA-seq data was confirmed by qRT-PCR in podocytes isolated from a different cohort of young and aged mice showing increased transcript levels for *Nlrp3* and key pathway mediators, *Casp1* and *Pycard* (Figure 1A). Next, to determine if the NLRP3 inflammasome was increased in middle-aged mice, we compared podocyte transcriptomes of young 4-month-old mice to middle-aged mice 19.5 months-old (~60 human years) (Supplementary Figure 1A). Gene set enrichment analysis (GSEA) again identified a signature consistent with an inflammatory phenotype (Supplementary Figure 1B). This includes pathways of the inflammatory response (Figure 1B), IL2/Stat5 signaling, IL6 signaling, interferon gamma response, allograft rejection, complement, and TNF signaling (Supplementary Figure 1B).

Immunostaining for NLRP3 in glomeruli across the mouse lifespan showed no signal at 4 and 12 months of age, but a progressive increase from 18 months of age (referred to herein as middle-aged) through 24 and 27- months-old (referred to as aged) (Figure 1D–1H). As expected, staining was absent in middle-aged NLRP3 null mice (Figure 1I). Glomerular immunostaining from 18 months of age was predominantly in a podocyte distribution, with the increase confirmed by quantification using a computer-assisted machine learning (Figure 1J). Weak NLRP3 staining was also detected in parietal epithelial cells and tubular epithelial cells from middle-aged mice that was markedly increased in aged animals. Aged (24 months) *Nphs1*-EGFP podocyte reporter mice confirmed the increase in NLRP3 in a different mouse strain, which co-localized with the EGFP reporter in podocytes (Figure 1K).

Next, to address the translational potential, we examined NLRP3 immunostaining in podocytes of aged human kidneys. NLRP3 was not detected in glomeruli from young (23 years old) healthy human kidneys (Figure 1L, 1M). In contrast, NLRP3 staining was increased in glomeruli of aged (82 years old) human kidneys (Figure 1N, 1O). Higher transcript expression levels for NLRP3 in micro-dissected human glomeruli were associated with increased percent of globally sclerosed glomeruli (Figure 1P), higher glomerular volume (Figure 1Q) and reduced podocyte density (Figure 1R), assessed as previously described [19, 38].

**Figure 2:**
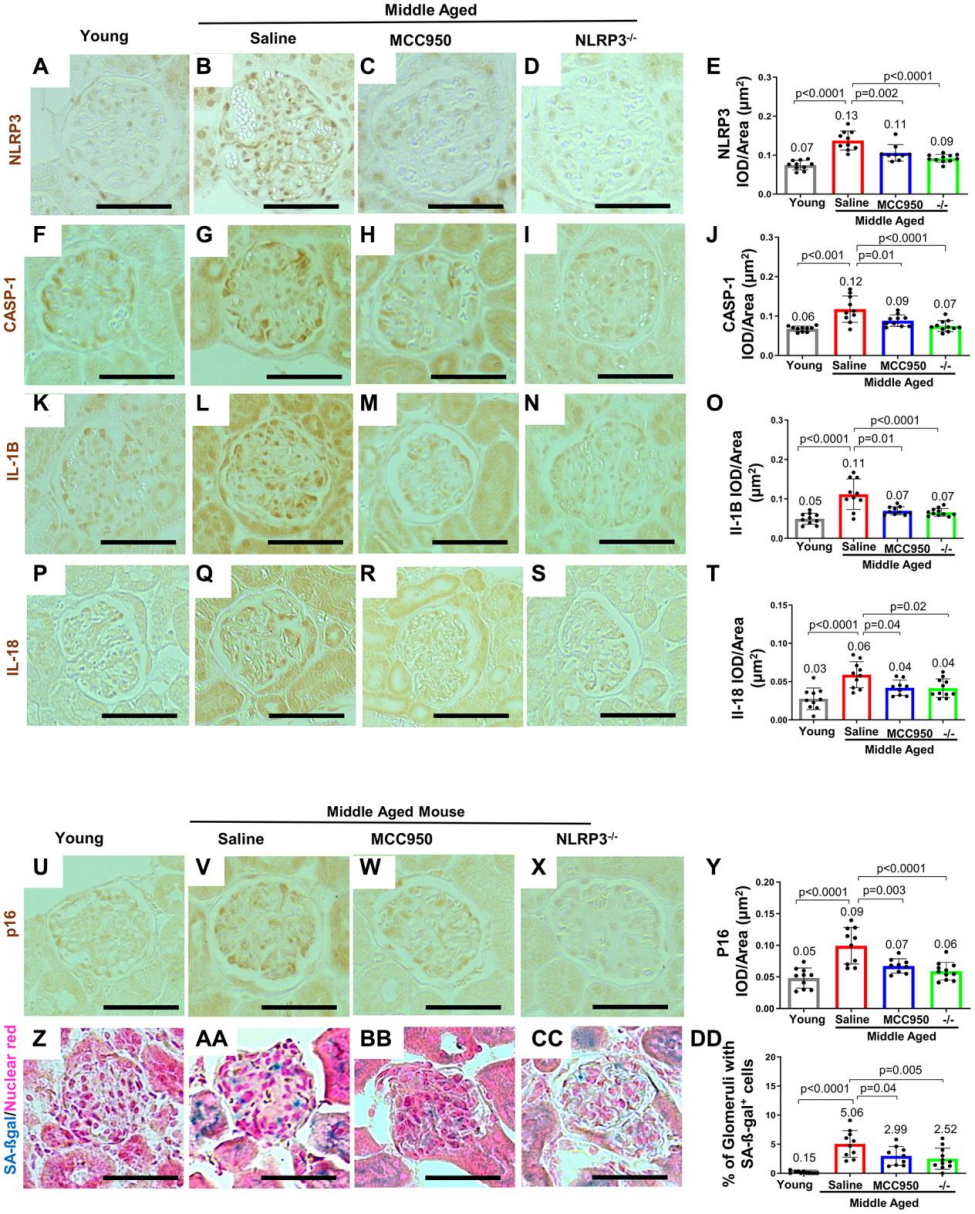
**Inhibiting/Deleting NLRP3 in middle-aged mouse podocytes.** (**A**–**Y**) Comparison of glomeruli from young and middle-aged mice treated with vehicle (Saline) or MCC950, as well as middle-aged Nlrp3-null mice by immunohistochemistry for NLRP3 (**A**–**E**), inflammasome downstream signaling components Caspase-1 (**F**–**J**), Interleukin-1β (IL-1β) (**K**–**O**), IL-18 (**P**–**T**) as well as the senescence marker p16 (**U**–**Y**). All immunostainings (brown color) were quantified using automated approaches, depicted as integral optical density/area (IOD/area) and analyzed by Student’s *t*-test statistics. (**Z**–**DD**) Staining for SA-β-Gal (blue) and nuclear red (red). SA-β-Gal is not detected in young mice (**Z**, **DD**), but increased in the glomerulus in saline treated middle aged mice, including podocytes (**AA**, **DD**). SA-β-Gal was lowered by MCC950 (**BB**, **DD**) and in age-matched NLRP3 null mice (**CC**, **DD**). Note that in all the conditions staining increased in glomeruli of middle-aged saline-treated and was lower in MCC950-treated (**C**, **E**) and in aged-matched NLRP3 null mice. The scale bars in the images correspond to 25 μm.

Finally, we examined key NLRP3 pathway mediators. In line with the mRNA-seq and the qRT-PCR analyses (Figure 1A–1C), immunostaining for NLRP3 (Figure 2A, 2B, 2E), Caspase-1 (Figure 2F, 2G, 2J), IL-1β (Figure 2K, 2L, 2O) and IL-18 (Figure 2P, 2Q, 2T) were higher in glomeruli of middle-aged mice compared to young mice, predominantly in a podocyte distribution. Similarly, Caspase-1 staining was barely detected in young healthy human kidneys but increased in podocytes with age (Supplementary Figure 2A–2D).

Together, these data are consistent with an increase in the NLRP3 inflammasome and downstream mediators in mouse and human podocytes in middle-aged and aged kidneys.

### NLRP3 expression is augmented in aged mice with experimental FSGS

Studies have shown an increase in NLRP3 in injured podocytes in disease. [23–37] This was confirmed in our experimental model of podocyte depletion induced FSGS, which showed an increase in NLRP3 staining (Supplementary Figure 3A, 3B). To determine if NLRP3 was further increased in aged mice with disease, experimental FSGS was induced in mice 24 months of age with a cytopathic anti-podocyte antibody. NLRP3 staining in the glomerulus was higher in aged mice with FSGS compared to age-matched mice without disease (Supplementary Figure 3C, 3D). These data support the notion that aging, and injury compound the increase in NLRP3 in podocytes.

### Pharmacological and genetic approaches to reduce NLRP3 in podocytes from middle-aged mice

To address the biological role of an increased NLRP3 inflammasome in middle-aged podocytes we used both pharmacological and genetic approaches. 18-month-old C57B6 mice were randomized to receive 6 weeks of either the NLRP3 inhibitor MCC950 [39], or vehicle (saline) (Supplementary Figure 4A). MCC950 treatment had no impact on body weight (Supplementary Figure 4B). As previously reported [19] clinical measurements of kidney function, Albumin: Creatinine Ratio (ACR), Blood Urea Nitrogen (BUN) and Soluble Urokinase Plasminogen Activator Receptor (suPAR), were not increased in control middle-aged mice and were therefore not impacted by reducing NLRP3 pharmacologically (Supplementary Figure 4C–4E).

Immunostaining demonstrated that MCC950 treatment was effective when compared to vehicle-treated littermates by reducing podocyte staining for NLRP3 (Figure 2B, 2C, 2E) and its downstream targets Caspase-1 (Figure 2G, 2H, 2J), IL-1β (Figure 2L, 2M, 2O) and IL-18 (Figure 2Q, 2R, 2T). These changes were similar to those seen in middle-aged NLRP3 null mice, where staining for NLRP3 (Figure 2D, 2E), Caspase-1 (Figure 2I, 2J), IL-1β staining (Figure 2N, 2O) and IL-18 (Figure 2S, 2T) were not detected in podocytes. These results show that MCC950 treatment and the genetic deletion of NLRP3 reduce the NLRP3 inflammasome and downstream mediators in middle-aged mice.

### Podocyte lifespan and glomerular ultrastructure were improved when NLRP3 was inhibited or absent

We next addressed whether NLRP3 inhibition in wildtype mice and/or genetic deletion of NLRP3 halted or even reversed podocyte aging in middle-age. As expected, compared to young mice, staining for the senescent activator gene p16^Ink4^ was higher in both glomerular epithelial cells and tubular epithelial cells of control middle-aged mice (Figure 2U, 2V, 2Y). Glomerular staining for p16 was predominantly localized to podocytes. Both MCC950 treatment and gene deletion in NLRP3 null mice resulted in reduced p16 staining compared to aged-matched saline and wildtype controls, respectively (Figure 2W, 2X). Analysis of senescence-associated beta-galactosidase (SA-β-gal) as a marker of aging showed similar results (Figure 2Z–2DD).

**Figure 3:**
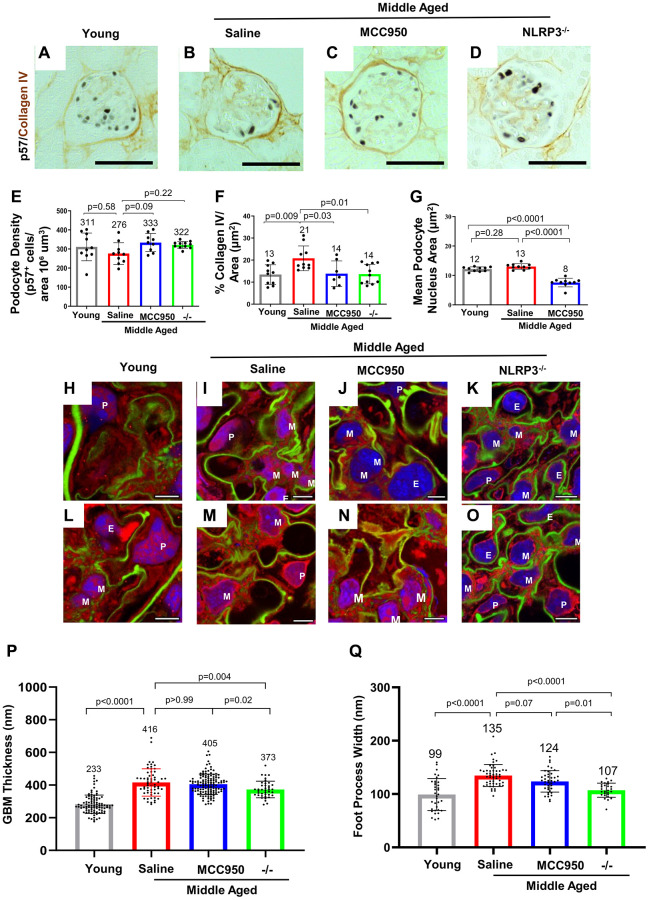
**Podocyte lifespan and glomerular ultrastructure were improved by inhibiting and deleting NLRP3.** (**A**–**F**) Immunostaining for p57 (black, nuclear) and Collagen IV (brown) comparing glomeruli of young, middle-aged saline-treated, MCC950-treated mice as well as NLRP3 null mice. Quantification of the number of p57-positive podocytes was used to determine podocyte density (**E**), Collagen IV staining to assess glomerular scaring (**F**) and their nuclear size was used to determine hypertrophy (**G**). Samples were compared using Student’s *t*-test and significance is indicated. Representative images are shown; the scale bars in the images correspond to 25 μm. (**H**–**O**) Analysis of glomerular ultrastructure using FLARE coupled to confocal microscopy; hydrogel-expanded mouse kidney tissue has been stained for primary amines (red, labels proteins), oxidized carbohydrates (green, labels basement membrane and mesangial matrix), and DNA (blue, labels nuclei). Representative images comparing glomeruli of young, middle-aged saline-treated, MCC950-treated mice as well as NLRP3 null mice are shown. All scale bars correspond to 5 μm pre-expansion. Abbreviations: P: podocyte; E: endothelial cells; M: mesangial cells. (**P**, **Q**) Quantification of glomerular basement membrane (GBM) thickness by measuring the average thickness of the oxidized carbohydrate stain in capillary loops (**P**) and of foot process width by determining the average thickness of the amine-stained foot processes at half-maximum (**Q**).

A major hallmark of glomerular aging is the decrease in the podocyte’s lifespan, which is characterized by a decrease in podocyte density [17, 19, 40–42]. Podocyte number was measured by staining for p57 (*Cdkn1c*) and quantitated with computer-assisted machine learning. Podocyte density decreased in saline-treated middle-aged vs. young mice (Figure 3A, 3B, 3E). Podocyte density was restored in glomeruli of middle-aged mice treated with MCC950 and middle-aged NLRP3 null mice (Figure 3C–3E). The improvement in podocyte lifespan in the MCC950-treated and the NLRP3 null mice was accompanied by reduced glomerular scarring as measured by Collagen IV immunostaining (Figure 3A–3D, 3F). Measuring nuclear size as a surrogate for podocyte hypertrophy [43–46] demonstrated a reduction in MCC950-treated podocytes (Figure 3G). The reversal of the aged podocyte phenotype was not caused by podocytes re-entering the cell cycle as the proliferation marker Ki-67 was not detected in podocytes of any mouse group (not shown).

Finally, we assessed glomerular ultrastructure by FLARE and expansion microscopy (Figure 3H–3O). Quantification of these images demonstrated that the glomerular basement membrane (GBM) thickness increased (283 nm vs. 416 nm, *P* < 0.0001, Figure 3P) and foot process width increased (107 nm vs. 134.7 nm, *P* < 0.0001, Figure 3Q) in middle-aged saline-treated control mice compared to young mice. While GBM thickness did not change during the 6-week-long MCC950 treatment (405 nm vs. 416 nm, *P* = 0.37), it was reduced in the middle-aged NLRP3 null mice (373 nm vs. 416 nm, *P* = 0.002, Figure 3P). This is in line with the idea that GBM remodeling is not instantaneous [47–49] and likely takes longer than the 6 weeks of MCC950 treatment. However, foot process width was reduced in both MCC950-treated mice (123.6 nm vs. 134.7 nm, *P* = 0.012) and in NLRP3 null mice (107.1 nm vs. 134.7 nm, *P* < 0.001, Figure 3Q). In summary, the data demonstrate that decreasing or deleting NLRP3 improved podocyte lifespan in glomeruli of middle-aged mice.

### Podocyte health was improved when NLRP3 was inhibited or absent

**Figure 4:**
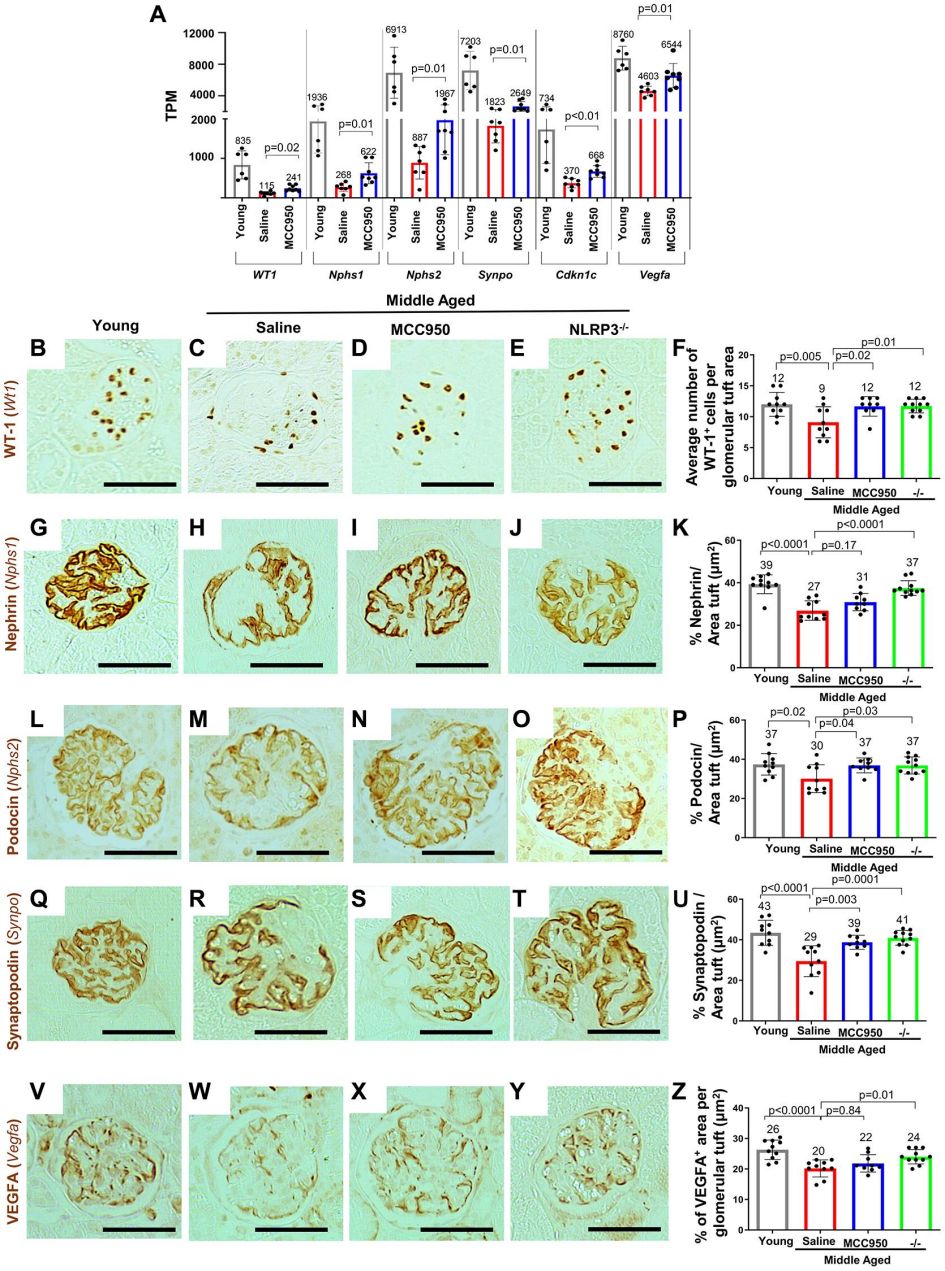
**Podocyte health was improved upon inhibition/absence of NLRP3.** (**A**) Comparison of the transcripts for canonical podocyte genes (*Wt1*, *Nphs1*, *Nphs2*, *Synpo*, *Cdkn1c*, *Vegfa*) using the normalized mRNA-seq read counts from isolated podocytes of middle-aged saline-treated (red bars) and MCC950-treated mice (blue bars) compared to their young counterparts (grey bars). (**B**–**Z**) Immunostaining of kidneys from young, saline- and MCC95-treated middle-aged as well as middle-aged Nlrp3 null mice for WT-1/Wt1 (**B**–**F**), Nephrin/Nphs1 (**G**–**K**), Podocyin/Nphs2 (**L**–**P**), Synaptopodin/Synpo (**Q**–**U**) and VEGFA (**V**–**Z**). Representative images are shown; scale bars represent 25 μm. Staining intensities were quantified and depicted in the graphs as % tuft area.

The podocyte’s health-span decreases with advancing age and is characterized by changes to their molecular, cellular and transcriptional landscape required for their normal physiology, structure and function [15]. Thus, to understand whether the NLRP3 inflammasome contributes to this aspect of podocyte aging and how the NLRP3 pathway impacts podocyte health-span, we initially focused on canonical podocyte genes essential for the highly specialized structure and function of these terminally differentiated cells. Compared to middle-aged/vehicle-treated mice, middle-aged/MCC950-treated mice had higher transcript levels for podocyte health-span genes such as *Wt1* (*P* = 0.02), *Nphs1* (*P* = 0.01), *Nphs2* (*P* = 0.01), *Synpo* (*P* = 0.01) and *Cdkn1c* (*P* < 0.01) (Figure 4A). Higher podocyte protein expression was confirmed by immunostaining for Wilms tumor protein (*Wt1*), Nephrin (*Nphs1*), Podocin (*Nphs2*) and Synaptopodin (*Synpo*) in both middle-aged/MCC950-treated and middle-agedNLRP3 null mice compared to middle-aged/vehicle-treated mice (Figure 4B–4U).

Finally, we analyzed vascular endothelial growth factor (VEGF) which is critical for maintenance of podocyte/endothelial cell interactions [50], as a surrogate for podocyte synthetic function. *Vegfa* mRNA levels decreased in middle-aged/vehicle-treated mice but were partially restored in podocytes from middle-aged/MCC950-treated (*P* = 0.01) (Figure 4A). This was partially validated at the protein level by immunostaining for VEGFa, with a trend toward increased staining in the middle-aged/MCC950-treated mice and a significant increase in the middle-aged NLRP3 null mice (Figure 4V–4Z). Together, these results show that lowering/deleting NLRP3 improved podocyte health-span in middle-aged mice.

### Transcriptomic changes in middle-aged podocytes regulated by the NLRP3 inflammasome

**Figure 5:**
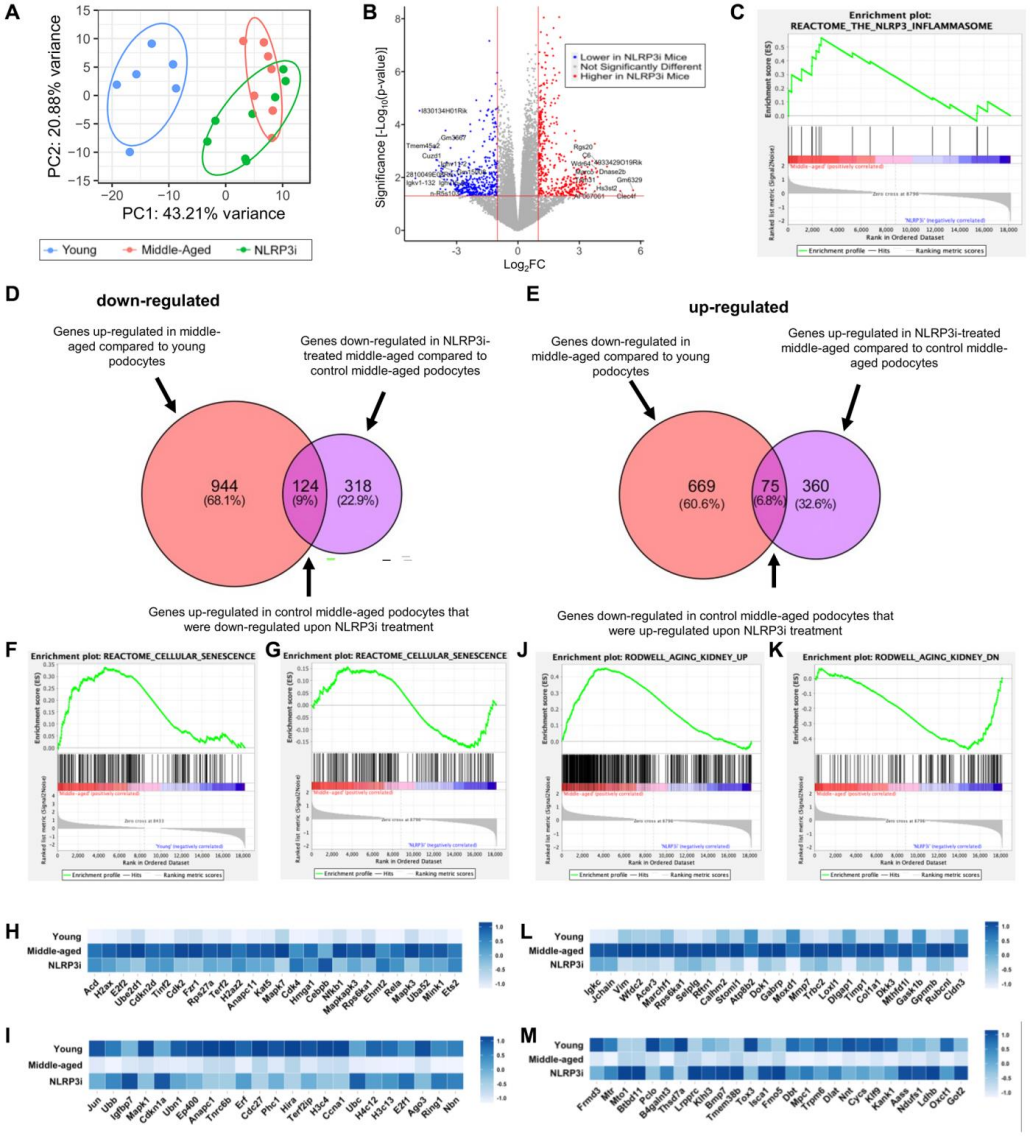
**Transcriptomic analysis of NLRP3 inhibition.** (**A**) Principal component analysis of the individual samples (young, middle-aged and middle-aged treated with MCC950 (NLRP3i)). (**B**) Volcano Plot comparing the saline- and MCC950-treated middle-aged podocyte transcriptomes; transcripts changed >2 and with a *p*-value > 0.05 are indicated in blue when down regulated upon NLRP3i and in red, when increased. (**C**) GSEA plot of the NLRP3 inflammasome gene set decreased upon treatment with MCC950. (**D**, **E**) Venn diagrams comparing the transcripts up-regulated in middle-aged podocyte and down-regulated upon NLRPi (**D**) and those down-regulated in the middle-aged and up-regulated upon NLRP3i (**E**) using the differentially expressed genes determined by the DSEQ2 analysis. (**F**–**I**) GSEA plots for the Reactome gene set cellular senescence comparing young to middle-aged (**F**) and middle-aged to middle-aged to NLRP3i-treated (**G**). Most differentially up- and down-regulated transcripts are compared along all three conditions in the histograms in panel H and I, respectively. (**J**–**M**) GSEA plots for the Rodwell aging kidney up (**J**) and down (**I**) gene sets comparing middle-aged to middle-aged to NLRP3i-treated. Most differentially expressed transcripts for either gene set are compared along all three conditions in the histograms (**L**, **M**).

To obtain a better understanding of potential mechanisms responsible for the slowing of glomerular aging upon inhibition of NLRP3, we isolated podocytes from individual mice from each group (young, middle-aged/vehicle-treated and middle-aged/MCC950-treated) and performed transcriptome analyses. Principal component analysis showed excellent clustering of the individual groups (Figure 5A) with many differentially expressed transcripts between middle-aged/MCC950-treated vs. middle-aged/vehicle-treated podocytes (Figure 5B). As expected, GSEA analysis demonstrated that podocytes from mice treated with MCC-950 exhibited inhibition of the NLRP3 signaling pathway (Figure 5C). We next analyzed the significantly altered transcripts for their contribution to the aging process. Using a threshold of 2-fold and a *P*-value of 0.05 a total of 442 genes were down-regulated and 435 genes were up-regulated in middle-aged/MCC950-treated podocytes compared to middle-aged/vehicle-treated podocytes (Figure 5D, 5E). Of the down-regulated transcripts, 124 genes (9%) were also up-regulated in aged vs. young podocytes. Similarly, of the up-regulated transcripts, 75 genes (7%) were down-regulated in aged vs. young podocytes. GSEA analysis (Supplementary Figure 1B) confirmed the data shown in Figure 2 at the global level where both up- and down-regulated genes in response to senescence were restored by NLRP3i treatment (Figure 5F–5I). These findings were extended by examining a gene set specifically generated to assess kidney aging [51] which showed an even more pronounced impact (Figure 5J–5M). In summary, these data suggest activation of NLRP3 signaling is part of the natural podocyte aging process.

### Age-regulated inflammation and podocyte signaling were restored by inhibiting NLRP3

**Figure 6:**
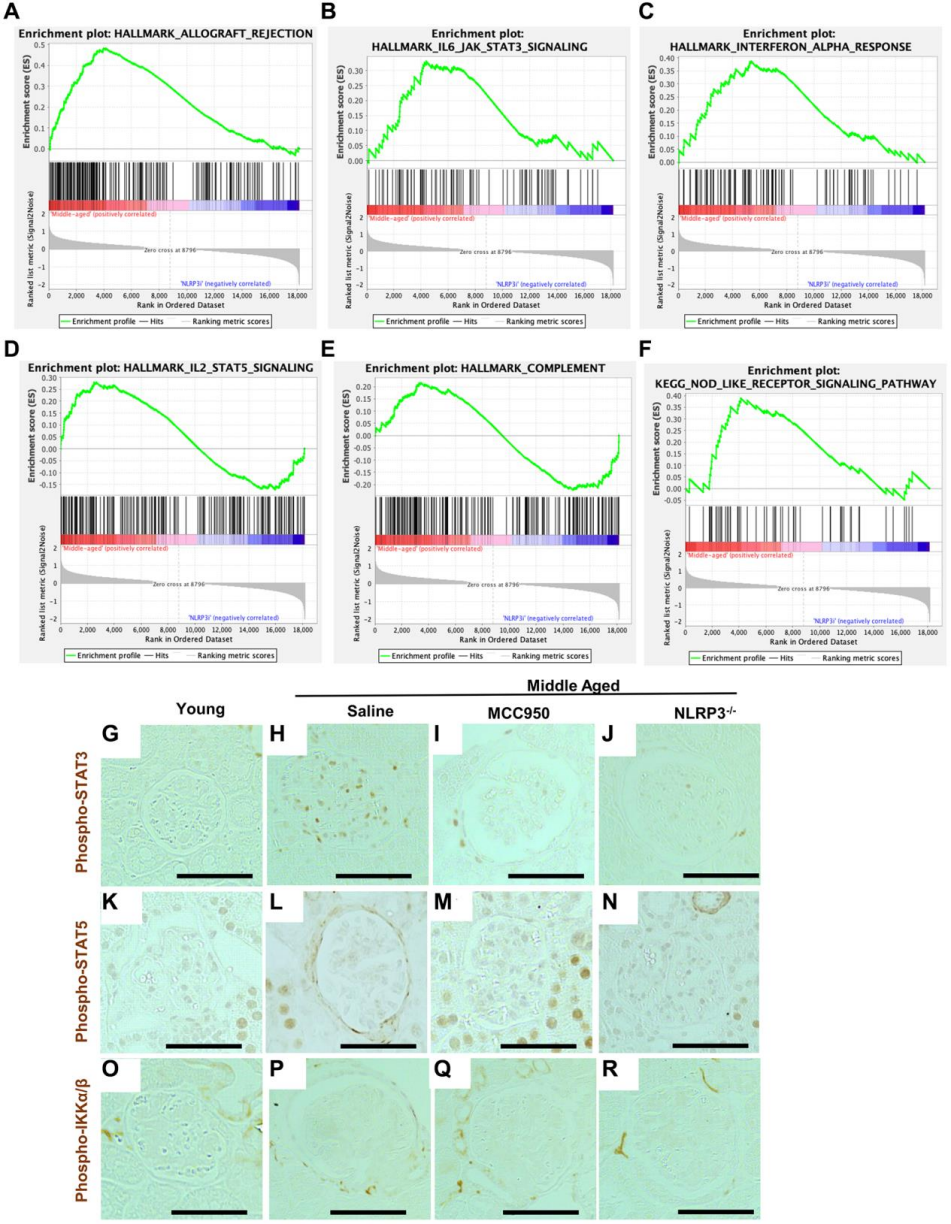
**Podocyte inflammaging is reduced by inhibiting NLRP3.** (**A**–**F**) GSEA plots comparing the transcriptomic data from saline- and MCC950-treaded middle-aged mice for 5 hallmark gene sets (allograft rejection, IL-6/JAK/STAT3 signaling, interferon alpha response, IL-2/STAT5 signaling and complement) as well as one KEGG gene set (Nod-like receptor signaling pathway). (**G**–**R**) Immunoperoxidase staining comparing glomeruli from young mice, to middle-aged either treated with saline or MCC950 as well as middle-aged NLRP3 null mice using phospho-STAT3 (**G**–**J**), Phospho-STAT5 (**K**–**N**) or Phospho-IKKa/β (**O**–**R**). Representative images are shown; scale bars in the images correspond to 25 μm. Note that in line with the GSEA data phospho-STAT3 staining was strongly reduced by interfering with NLRP3 signaling in middle-aged mice, while STAT5 showed a more modest change.

Interestingly, GSEA showed that inhibition of NLRP3 signaling was consistent with the reversal of the inflammatory phenotype in podocytes (Supplementary Figure 1C). In addition to the NLRP3 inflammasome, several additional inflammatory pathways that were increased in middle-aged podocytes compared to young were at least partially lowered in mice treated with MCC950. This included allograft rejection (Figure 6A), IL6 signaling (Figure 6B), Interferon alpha (Figure 6C), IL2/Stat5 signaling (Figure 6D), complement (Figure 6E) and NOD-like receptor signaling (Figure 6F). This effect was confirmed by immunostaining for phospho-STAT3, a marker of active interferon and IL6 pathways (Figure 6G–6J). Phospho-STAT3 immunostaining was not detected in podocytes of young mice, was increased in middle-aged saline-treated mice, but was reduced by MCC-950 treatment and was barely detected in the podocytes of NLRP3 null mice. A similar trend was observed for phospho-STAT5, a marker for active IL2 signaling (Figure 6K–6N) Importantly, not all inflammatory pathways were increased in middle-aged saline-treated mice nor reduced following NLRP3 inhibition. For example, immunostaining for phospho-IKKa/β, a marker of active TNF signaling, did not increase in middle-aged glomeruli (Figure 6O–6R).

### NLRP3 inhibition does not majorly impact apoptosis, endoplasmic reticulum stress or autophagy

**Figure 7:**
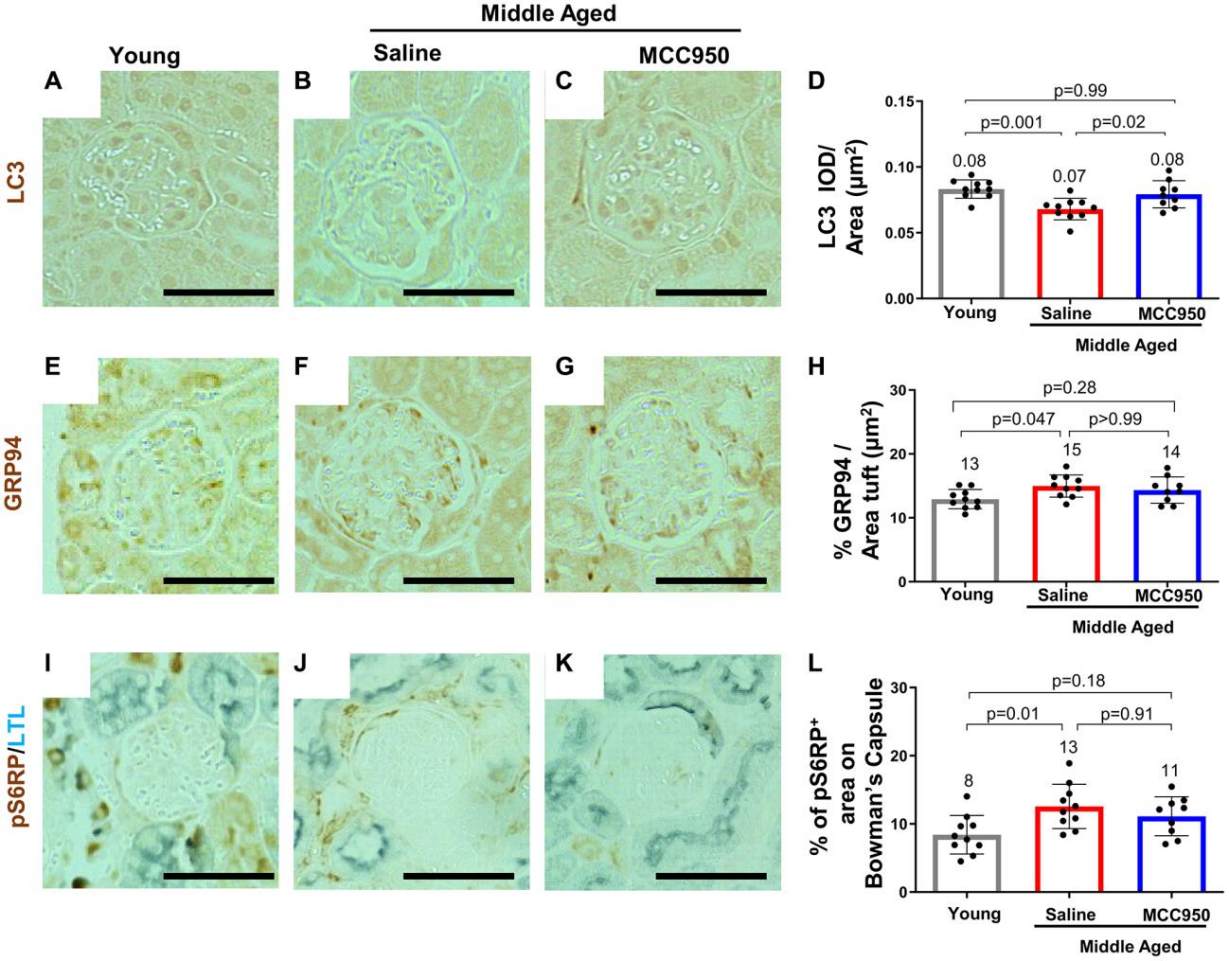
**MCC950 treatment improves autophagy signaling.** Immunostaining for autophagy marker LC3 (**A**–**D**), for the ER stress response protein GRP94 (**E**–**H**) and the mTOR signaling readout pS6RP (**I**–**L**) comparing young to middle-aged saline- and MCC950-treated mice (**I**–**L**). Panels **I**–**K** were counterstained with Lotus Tetragonolobus Lectin (LTL, blue) to visualize proximal tubules. Abbreviation: BC: Bowman’s Capsule. Images were quantified and depicted as % tuft area Scale bars in the images correspond to 25 μm.

A reduction in canonical gene/protein expression in aged podocytes is normally accompanied by cell death, detachment and an inability of podocytes to self-renew to replace the lost cells [52]. Interestingly, besides the KRAS hallmark pathway, which has been shown to be functionally connected to NLRP3 signaling, [53, 54] none of those pathways were identified by the GSEA (Supplementary Figure 1B). To further substantiate this observation, we performed immunofluorescence studies comparing glomeruli of young, saline- and MCC950-treated middle-aged mice. Indeed, apoptosis as a contributor to podocyte death seems unlikely, as we were unable to detect increased glomerular Caspase-3 staining in control middle-aged mice (not shown). Other pathways shown to lead to podocyte loss [52] such as the endoplasmic reticulum stress (ERS) [55, 56] and autophagy stress pathways were also only slightly or not at all impacted by NLRP3i treatment. Immunostaining for the autophagy marker LC3 was barely detected (Figure 7A–7D). Conversely, the ERS associated proteins GRP94/Hsp90b1 were up-regulated in the middle-aged mice, but not significantly restored by MCC950 treatment (Figure 7E–7H). Finally, the active mTOR marker pS6 ribosomal protein (pS6RP) was higher in saline treated middle-aged mice but was unaffected by MCC950 treatment (Figure 7I–7L). Together, these data suggest that the pro-podocyte effect of NLRP3 inhibition at middle-age was primarily caused by inhibition of pyroptosis and only to a small extent by reduced ER stress and augmented autophagy.

### Impact of NLRP3 inhibition on non-podocyte glomerular cells

**Figure 8:**
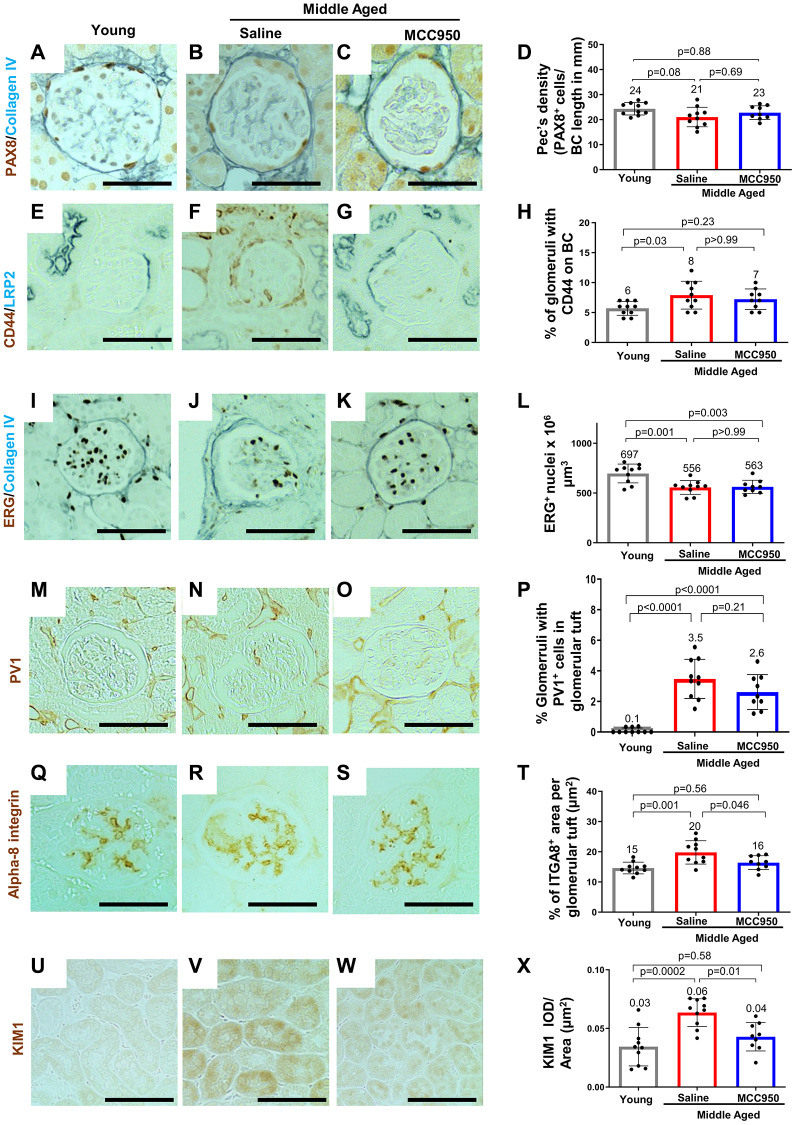
**Impact of MCC950 treatment on other glomerular cells and proximal tubules.** Immunostainings comparing young to middle-aged saline- and MCC950-treated mice. (**A**–**D**) PAX8 (brown, nuclear) and Collagen IV staining (blue) was used to visualize parietal epithelial cells (PECs) and Bowman’s capsule, respectively. Data were quantified and PEC density was calculated by the ratio of PAX8-positive cells and Bowman’s capsule (BC) length (**D**). (**E**–**H**) Immunostaining for CD44 (brown) and LRP2 (blue) was used to label activated PECs and proximal tubules, respectively (**E**–**G**). To determine the number of glomeruli with activated PECs, data were quantified based on the presence of CD44 staining in BC (**H**). (**I**–**L**) Endothelial cell number was determined staining with the endothelial cell marker ERG (nuclear brown staining) and the BC marker Collagen IV (blue) (**I**–**K**). Data were quantified as number of nuclei per mm^2^ (**L**). (**M**–**P**) To assess endothelial injury, kidney sections were stained for PV-1 (brown) as a marker of injured endothelial cells (brown) and quantified as percentage of glomeruli with PV-1-positive cells (**P**). (**Q**–**T**) Alpha-8 integrin (ITGA8) staining (brown) was used as a measure of the mesangial area (brown) (**Q**–**S**) and quantified as the percent ITGA8-positive area within the glomerular tuft (**T**). (**U**–**X**) To determine the effect of MCC950 on proximal tubules, sections were stained with KIM1 (brown) (**U**–**W**) and quantified as IOD per area (**X**). In all panels representative images are shown and scale bars in the images correspond to 25 μm. In all the graphs, error bars are standard deviation, and the mean levels are stated by the number above the bars.

Compared to young mice, middle-aged/vehicle-treated mice had a decrease in the density of parietal epithelial cells (PAX8 staining) (Figure 8A–8D) accompanied by an increase in their activation (CD44 staining) (Figure 8E–8H). Glomerular endothelial cell density (ETS-related gene, ERG [57] staining) was also decreased in middle-aged/vehicle-treated mice (Figure 8I–8L), accompanied by an increase in the glomerular endothelial injury marker plasmalemmal vesicle associated protein-1 (PV1) [58] (Figure 8M–8P). The mesangial marker alpha 8 integrin was higher in middle-aged/vehicle-treated mice compared to young mice (Figure 8Q–8T). However, in contrast to the data shown earlier on middle-aged podocytes, none of the changes in non-podocyte glomerular cells were impacted by inhibiting NLRP3 with MCC950 (Figure 8). In contrast, the increase in the tubular epithelial cell injury marker KIM1 was reduced in middle-aged/MCC950-treated mice (Figure 8U–8X).

### NLRP3 signaling is increased in injured human kidney organoids

To address whether inhibiting NLRP3 signaling improves podocyte injury and if NLRP3 increases senescent inducing genes in humans, we used a human kidney organoid model. Day 14 human kidney organoids were exposed to cytopathic anti-podocyte antibody with and without NRLP3 inflammasome inhibitor, MCC950. Induction of experimental FSGS caused upregulation of NRLP3 transcription along with other canonical NRLP3 inflammasome and senescence markers such as IL-1β, CASP1, PYCARD and CDKN2D (Supplementary Figure 5). Concurrent treatment with MCC950 resulted in a decreased expression of the NRLP3 inflammasome markers and CDKN2D (Supplementary Figure 5). These data support the notion that injury compounds the increase in NLRP3 and senescence in podocytes.

### NLRP3 signaling in liver aging

We have previously shown that inhibition of PD1 signaling improves not only podocyte aging but also has a beneficial effect on liver aging [19]. Thus, we wondered whether lowering NLRP3 would have a similar anti-aging effect in the liver. As in the kidney, the age-associated increase in the NLRP3 target IL-1β was reduced by MCC950, consistent with NLRP3 inhibition (Supplementary Figure 6A–6C, 6E). This was accompanied by a decrease in the characteristic fat deposition in aging liver visualized using Oil Red O staining (Supplementary Figure 6F–6H, 6J). The effects from the MCC950 treatment were confirmed in middle-aged NLRP3 null mice (Supplementary Figure 6D, 6E, 6I, 6J). Together, these data suggest that the beneficial consequences of inhibition of NLRP3 signaling were not restricted to the kidney and that systemic NLRP3 inhibition has a more widespread anti-aging effect.

## DISCUSSION

The current study examined changes in podocytes of middle-aged mice (19.5 months age ~60 human years) to determine if interfering with critical inflammatory pathways might slow healthy glomerular aging. We previously reported that the NLRP3 inflammasome was increased in podocytes of aged mice (24-month-old, ~70+ human years) [20]. Here, we showed NLRP3 was already increased by middle-age in mice, and in podocytes of kidneys from middle-aged healthy humans. This upregulation was functionally relevant and caused upregulation of inflammasome signaling, as the NLRP3 effector protein Caspase-1 and Caspase-1-dependent pro-inflammatory cytokines Interleukin-1β and Interleukin-18 were also increased. In sum, these findings are consistent with an age-induced increase in NLRP3 inflammasome signaling in podocytes by middle-age. Moreover, this study shows that similar to many forms of podocyte injury, [23–37] aging and injury are both associated with increased NLRP3 expression.

Additional inflammatory pathways including IL2/Stat5 signaling, IL6 signaling, interferon gamma, allograft rejection, complement, and TNF signaling were also increased. Together, these data support the existence of an inflammatory phenotype in podocytes by middle-age that, as we have reported, persists with further aging [20]. Human kidney tissue showed significant associations of higher glomerular transcript expression of NLRP3 with higher global glomerulosclerosis, glomerular hypertrophy and reduced podocyte density which supports this further. This is functionally important because reducing NLRP3 signaling pharmacologically in middle-aged mice and in human kidney organoids, and by gene deletion in mice, decreases podocyte senescence and aging.

Pyroptosis via NLRP3 and Caspase-1 was originally described as an antimicrobial response following infection by intracellular pathogens [59]. Yet, it has also been reported in epithelial cells, including podocytes [52], as well as in chronic diseases [59]. Our data add two important observations to inflammasome signaling in podocytes: First, podocytes undergo pyroptosis as early as middle-age, when there is a decline in podocyte density and increasing glomerular scarring. As such it may be a critical contributor to the lower threshold for injury (e.g., glomerular disease, hypertension, diabetes, and reduction in nephron number such as nephrectomy or organ donation) in this age group. Second, the activation of NLRP3 signaling in podocytes resembles inflammaging, a process defined as a chronic, low-grade sterile inflammation with advancing age in the absence of overt infection [60].

To address the functional implications of these observations, we asked whether limiting NLRP3 signaling in middle-aged mice could slow or even reverse podocyte pyroptosis and/or inflammaging. A short, 6-week pharmacological treatment with the selective NLRP3 inflammasome inhibitor MCC950 [39], and a genetic approach using NLRP3 null mice both reduced podocyte pyroptosis in middle-aged mice as evidenced by a decrease in Caspase-1, IL-1β and IL-18 inflammasome pathways. The effect also decreased other inflammatory pathways such as allograft rejection, IL6-JAK-stat, IFNalpha, IL2-Stat5 and complement.

More impressively, our data suggest that the age-dependent increase in NLRP3 signaling promotes podocyte senescence/aging in middle-aged mice. Inhibiting and/or deleting NLRP3 caused (a) a decrease in senescent associated β-Galactosidase (SAβ-gal) staining, (b) a reduced expression in senescent inducing p16, (c) global transcriptomic repression of several SASP mRNAs and (d) a reversal of genes present in the Rodwell Aging Kidney gene set [51]. Thus, in addition to the NLRP3-inflammasome playing an important role in podocyte injury in glomerular diseases [21, 29, 61–63], we believe this is the first report of its role in podocyte senescence and aging. Moreover, by showing that in aged mice with experimental FSGS, injury augments the age-dependent increase of NLRP3 signaling, it makes this pathway an intriguing therapeutic target for podocyte diseases in the elderly. Finally, reducing NLRP3 signaling in human kidney organoids used as a second model also reduced the senescent gene CDKN2D.

A hallmark of podocyte aging is a decrease in cellular lifespan, defined as a progressive decrease in number and density with age [15]. Podocyte lifespan was higher in NLRP3 inhibited and null mice compared to age-matched control, which was accompanied by reduced glomerular collagen IV. Because podocytes are post-mitotic and we did not detect the proliferation marker Ki-67, we interpret these results to be consistent with the inhibition/deletion of NLRP3 reducing pyroptosis-associated podocyte loss. Caspase-3-associated apoptosis, the endoplasmic reticulum stress and autophagy stress pathways implicated in podocyte loss [55, 56] were only slightly or not at all impacted by MCC950 treatment and thus probably only be a minor contribution to the phenotype.

The aged podocyte phenotype is also characterized by a decrease in its health-span defined as the part of a podocyte’s life that is spent in good health and able to perform normal cellular and molecular functions to maintain their physiology, structure and function [15]. Surprisingly, inhibition of NLRP3 signaling also improved the health-span of middle-aged podocytes at multiple levels. Compared to vehicle treated mice exhibited (i) a higher expression of the canonical podocyte proteins (e.g., synaptopodin, podocin, and nephrin); (ii) a higher expression of podocyte transcription factors (e.g., WT1 and E2F), (iii) a higher level of *VEGFa* mRNA and protein used as a proxy for podocyte synthetic function, (iv) reduced hypertrophy, (v) reduced foot process width and (vi) reduced extracellular matrix production as shown by a decrease GBM thickness in the null mice. It is noteworthy that these changes were already observed after only 6 weeks of MCC950 treatment. In fact, most changes were like those seen in mice with genetic ablation of *Nlrp3*. The one exception are the morphometric parameters (i.e., foot process width and GBM thickness). These improved with MCC950 treatment but not to the same extent. Yet, these parameters are likely the most challenging to restore as ECM turnover is relatively slow [47–49]. Moreover, with the global *Nlrp3* knockout the changes may have never (or only slightly) occurred, whereas the MCC950 treatment was only commenced in middle aged mice where and aged phenotype was already present. In fact, the latter demonstrates that the effects of aging are modifiable and that there is a therapeutic window to counter them.

The parietal epithelial, endothelial and mesangial cells showed some, yet less pronounced changes in NLRP3 expression upon aging. Yet, in contrast to podocytes, MCC950 treatment had no detectable impact on the age-induced changes in these cell types. Tubular epithelial cell injury was also reduced by MCC950. These differences could be due to intrinsically different responses to NLRP3 activation or due differences in how these cells respond to age-dependent injury, as they in contrast to podocytes can proliferate and thus replace lost cells. Finally, it could be due to the timing of aging as it is thought that podocytes are the first glomerular cells undergoing aging and that they affect the remaining glomerular cells via paracrine signaling [15]. Thus, while podocytes are susceptible to the consequences of NLRP3 activation, the others may not yet be at middle age.

The effects of NLRP3 were clearly beyond only the kidney. In the liver, age-induced upregulation of IL-1β and Oil Red O were reduced in both MCC950-treated and Nlrp3 null middle-aged mice. This is in line with data demonstrating that MCC950 reduces liver inflammation and fibrosis in experimental NASH in mice [64], and the that the livers of aged NLRP3 null mice exhibit less fibrosis and oxidative damage compared to their younger counterparts [65]. Others have linked the NLRP3 inflammasome to senescence/aging [66] such as in the heart [67], endothelial cells [68], and ovaries [69]. Caloric restriction, an approach to prolong health-span, decreases the NLRP3 inflammasome [70]. These results, together with the data presented here, suggest NLRP3 signaling is a more generalized pathway involved in tissue aging.

We recognize some limitations in our study. As with any drug treatment, the administration of MCC950 might have been more impactful, if we used higher doses or a longer duration. Yet, the fact that even a relatively short 6-week treatment regimen had such a strong effect is promising. Second, the systemic nature of MCC950 administration and the global deletion of NLRP3 does not address whether the pro-aging effects of the NLRP3 inflammasome are coming from the podocytes only or whether they are due to a crosstalk with the other glomerular cell types. Third, this study only used male mice. Thus, sex as a biological variable was not addressed. We opted to focus on males, as kidney function declines earlier with aging compared to their female counterparts [71–73]. But obviously follow-up studies in females needs to be considered. Finally, we have not addressed the upstream regulators for the activation of NLRP3 signaling, i.e., how it becomes expressed and how it becomes activated. In cancer, KRAS activation has been shown to trigger inflammasome activation [53]. Interestingly, our GSEA data have shown changes in the KRAS signature and thus it is tempting to speculate that it is similarly involved in establishing or maintaining the NLRP3 expression in podocytes. Similarly, the mere presence of NLRP3 is insufficient to trigger signaling. A secondary stimulus is required. In addition to viral, bacterial, fungal, and protozoan pathogens this includes particulate matters, extracellular ATP, and toxins [74]. Whether there is a specific trigger in aging podocytes or whether this is simply an accumulation of insults will be an important area of future studies. In particular, as it may offer therapeutic avenues beyond the global inhibition of the NLRP3 inflammasome using MCC950 as used in this study or e.g., the STING inhibitor C-176 [75, 76]. Age-related GSK3β overexpression drives podocyte senescence, [18] and GSK3β promotes kidney damage through activation of the NLRP3 inflammasome [77]. Thus, one might speculate a possible role for NLRP3 in mediating the pro-senescent effect of GSK3β. Finally, the activation of endogenous viruses during aging have been shown in organ systems [78, 79], but have not yet been fully addressed in the aged kidney. While viruses can be detected in patients with kidney injury [80], their biological impact is still unknown.

In summary, our results demonstrate for the first time that aging podocytes acquire an inflammatory phenotype, which include the NLRP3 inflammasome and which is consistent with inflammaging. This occurs as early as middle-age (19.5 months-old mouse or ~60 human years) and contributes to decreased health- and lifespan of podocytes. Moreover, the beneficial effects of interfering with NLRP3 signaling makes targeting the inflammasome a valuable therapeutic target in particular in situations when nephron or podocytes numbers are already lower due to premature birth, genetic predisposition or aging and kidney function is challenged due to injury [15, 81].

## MATERIALS AND METHODS

### Animals

Experimental male C57BL/6J mice (WT, Strain #000664) aged 18 months (*n* = 20) were obtained from The Jackson Laboratory (Bar Harbor, ME, USA). Animals received 10 mg/kg MCC950 sodium (Cat# HY-12815A, MedChemExpress) in saline (Sodium chloride 0.9% Injectable, BD), or saline vehicle alone, via twice weekly IP injections, for 6 weeks. Control male C57BL/6J (WT, Strain #000664) mice aged 4 (*n* = 5), 12 (*n* = 5), 18 (*n* = 5), 24 (*n* = 5), and 27 (*n* = 5) months were from our lab animal tissue archives and originally obtained from The Jackson Laboratory (Bar Harbor, ME, USA). Breeding pairs to generate male B6.129S6-Nlrp3tm1Bhk/J (Nlrp3KO, Strain #021302) were obtained from The Jackson Laboratory then aged to 19.5 months (*n* = 11).

Experimental focal segmental glomerulosclerosis (FSGS) was induced in 4-month-old (*n* = 6) and 24-month-old (*n* = 4) wildtype male C57BL/6J mice (Strain #000664, The Jackson Laboratory) by two intra-peritoneal (IP) injections, 24 hours apart, of a cytopathic sheep anti-glomerular antibody (9 mg/20 g body weight) [82–84]. Kidney tissue was collected at baseline 4 m (*n* = 5) and 24 m (*n* = 5) and 28 days following antibody injection.

Nphs1-FLPo|FRT-EGFP double transgenic mice were bred in house to express FLP recombinase as previously described by Goldberg et al. [85], resulting in the FLP mediated excision of an FRT flanked STOP in the RCE: FRT [86] (FRT-EGFP, Strain #010812) mouse available from The Jackson Laboratory (Bar Harbor, ME, USA). Resulting animals have permanent EGFP expression in all podocytes, for the lifetime of the animal. There were 2 groups of Nphs1-EGFP reporter mice: young (4 months, *n* = 15) and aged (24 months, *n* = 24).

All mice were housed in the animal care facility at the University of Washington under specific pathogen-free conditions. All animals were co-housed in social groups of 2–5 animals, provided with environmental enrichment (nestlets), maintained on a 14-hour light/10-hour dark cycle, at 70 ± 5 F and 50 ± 10% relative humidity, with ad libitum access to food and water. Aging animals (over 18 months) received additional monitoring according to our aging mice protocols to ensure healthy aging. Mice were sacrificed at the ages described above by cervical dislocation according to American Veterinary Medical Association (AVMA) guidelines for the euthanasia of animals by certified personnel. Animal protocols were approved (2968-04) by the University of Washington Institutional Animal Care and Use Committee. Weights were taken bi-weekly for the duration of the study for animals receiving interventions and all animals were checked daily by vivarium personnel.

### Urine analysis

Urine was collected by pooling two spots on a single day at baseline and week 6 of MCC950 treatment. Albumin to creatinine ratio was calculated from urinary albumin measured by radial immunodiffusion assay (RID) as previously described [87] and creatinine measured by Creatinine (urinary) Colorimetric Assay Kit (Cayman Chemical, Ann Arbor, MI, USA).

### Blood urea nitrogen and suPAR

Blood was collected at sacrifice. Blood Urea Nitrogen was measured with a colorimetric Urea Assay Kit (Abcam, Cambridge, United Kingdom). Circulating suPAR levels were measured in plasma samples collected at sacrifice, using Mouse uPAR DuoSet ELISA (Cat, #DY531, R&D Systems Minneapolis, MN, USA).

### Immunostaining, quantification and visualization

Immunoperoxidase staining was performed on 4 μm thick formalin fixed paraffin-embedded (FFPE) mouse and human kidney sections as previously described [88]. Briefly, paraffin-embedded, formalin-fixed sections were incubated in Histoclear (National Diagnostics, Atlanta, GA, USA) and graded series of ethanol for rehydration. Sections were boiled in antigen retrieval buffer (Citric acid buffer pH 6.0 or EDTA buffer pH6.0, pH 8.0). To avoid nonspecific protein binding, Background Buster (Accurate Chemical and Scientific, Westbury, NY, USA) was used, as well as goat anti-rabbit Fab fragment and rabbit Fab fragment (Jackson ImmunoResearch Laboratories, West Grove, PA, USA). Suppression of endogenous biotin activity was performed using Avidin/Biotin Blocking Kit (Vector Laboratories, Burlingame, CA, USA). Initial primary antibodies were incubated overnight at 4°C. In the case of double immunostaining, subsequent primary antibodies were incubated either overnight at 4°C or for 3 hours at room temperature. Secondary antibodies and streptavidin conjugates were incubated for one hour at room temperature. The primary antibodies used in the study are summarized in Supplementary Table 1. Diaminobenzidine (Sigma-Aldrich, St. Louis, MO, USA) with or without 8% nickel chloride, was precipitated to visualize immunoperoxidase staining.

Immunofluorescence staining was performed on frozen sections that were thawed and washed in PBS. Gentle antigen retrieval was performed by warming citric acid buffer (pH 6.0) or EDTA buffer (pH 8.0) to 38°C. They underwent the same blocking and antibody incubation protocol as described for immunoperoxidase staining. All immunofluorescence samples were mounted using Vectashield mounting medium with 4′,6-diamidino-2-phenylindole (DAPI) (Vector Laboratories, Burlingame, CA, USA).

To quantify immunohistochemistry, slides were scanned in brightfield with a 20× objective using a NanoZoomer Digital Pathology System (Hamamatsu City, Japan). The digital images were imported into Visiopharm software (Hoersholm, Denmark) and its Image Analysis Deep Learning module was trained to detect glomeruli and assess immunohistochemical staining-positivity for Collagen IV, p57, Nephrin, Synaptopodin and ERG. The glomeruli ROIs were processed in batch mode generating per area outputs, cell counts and analyzed from 100% of the tissue sections. Quantification of podocin, VEGFA, NLRP3, IL-1B, LC3, GRP94, p16 and SA-b-galactidase staining was performed by using ImageJ 1.46r software (National Institutes of Health).

### Senescence-associated β-galactosidase (SA-β-Gal) staining

Frozen kidney sections (10 μm) were stained with SA-β-gal per kit instructions (Cell Signaling Kit #9860), then counter stained with nuclear fast red to determine senescence-associated-β-galactosidase [89, 90]. The percentage of glomeruli with SA-β-gal-positive staining in the glomerular tuft was quantified in outer cortex of each kidney section.

### Assessment of liver aging

Immunostaining was performed for Oil Red O (to detect lipids and triglycerides) as previously described [91]. To determine morphological changes present in aged liver, frozen liver sections (6 μm) were stained for Collagen IV and the endothelial marker CD31/PECAM-1 as previously described [92].

### FLARE staining and expansion microscopy

Kidney sections were cut to a thickness of 50 μm from cryo-preserved mouse kidney tissue blocks. Hydrogel-expansion and FLARE staining of kidney sections were performed according to the published FLARE protocol [93]. In brief, oxidized carbohydrates were labeled with ATTO 565 hydrazide, amines (proteins) were labeled with ATTO 647N NHS ester, and nuclei were labeled with the fluorescent DNA-binding dye SYBR Green I. Expanded samples were transferred onto a poly-L lysine coated coverslip (24 mm by 50 mm, no. 1.5; Fisher Scientific, #12544E) and imaged immediately. Images were acquired using a Nikon A1R inverted point-scanning confocal microscope at the University of Washington Biology Imaging Facility. A CFI Apo LWD Lambda S 40× objective lens with 1.15 numerical aperture was used on the microscope. Three-color 3D stacks were acquired with 130 nm lateral sampling and 570 nm axial sampling. The final FLARE images are red (amines), green (carbohydrates) and blue (DNA).

### Magnetic activated cell sorting (MACS) of podocyte and non-podocyte cell fractions

Kidney tissue (w/o the kidney capsule and surrounding fat) was placed into ice cold RPMI 1640 medium (w/o L-glutamine and phenol red, GE Healthcare Bio-Sciences, Pittsburgh, PA, USA). After removal of the medulla, the remaining cortex was minced into fine pieces and digested in 0.2 mg/ml Liberase™ TL (Sigma-Aldrich, St. Louis, MO, USA), 100 U/ml DNAse I (Sigma-Aldrich, St. Louis, MO, USA) in RPMI 1640 medium (w/o L-glutamine and phenol red) by shaking at 37°C for 30 minutes. The digest was passed through an 18G needle (Becton Dickenson, Franklin Lakes, NJ, USA) 10 times and enzymes were inactivated by adding 5 ml of RPMI 1640 medium (w/o L-glutamine and phenol red) supplemented with 1 mM sodium pyruvate (ThermoFisher Scientific, Waltham, MA, USA), 9% Nu-Serum™ IV Growth Medium Supplement (Corning Incorporated-Life Sciences, Durham, NC, USA) and 100 U/ml Penicillin-Streptomycin (ThermoFisher Scientific, Waltham, MA, USA). The cell suspension was passed through a 100 μm and a 40 μm cell strainer (BD Biosciences, San Jose, CA, USA) and pelleted by centrifugation at 200G at 4°C for 5 minutes. Cells were were resuspended in media containing two rabbit anti-Nephrin antibodies [94] (1:100, Abcam, Cambridge, MA, USA). After 1 hour at 4°C, cells were pelleted, washed in media and incubated with anti-rabbit microbeads (Miltenyi Biotec, Auburn, CA, USA) along with Alexa Fluor^®^ 594-conjugated AffiniPure Donkey Anti-Rabbit IgG 1:200 (in order to visualize binding of the Nephrin antibodies to the podocytes) for 30 minutes at 4°C. Cells were pelleted and washed in PBS with 0.5% BSA and 2 mM EDTA and applied to MACS LS columns (Miltenyi Biotec, Auburn, CA, USA) to gently separate microbead-bound podocytes from the other kidney cells. Cells not retained by the magnetic field were collected, pelleted and designated non-podocyte (NP) fractions. LS columns were removed from the magnetic field then washed with PBS with 0.5% BSA and 2 mM EDTA and podocytes were collected. A small aliquot of each fraction was imaged using an EVOS FL Cell Imaging System to verify podocyte isolation, based on the presence of Nephrin antibody. Additionally, qRT-PCR for a panel of podocyte genes was performed in both podocyte and non-podocyte fractions to confirm cell type identity.

### RNA isolation, qRT-PCR, library preparation and sequencing

mRNA was isolated using the RNeasy Mini Kit (Qiagen, Germantown, MD, USA) as per the manufacturer’s instructions and used for bulk mRNA sequencing or converted to cDNA by reverse transcription with the High-Capacity RNA-to-cDNA Kit (Thermo Fisher, Waltham, MA, USA) and utilized for quantitative real-time PCR analysis. qRT-PCR was performed using iTaq SYBR Green Supermix (Bio-Rad, Hercules, CA, USA) and a QuantStudio 6 Flex real-time PCR System (Applied Biosystems, Waltham, MA, USA) as we have previously described [20]. Relative mRNA expression levels were normalized to *glyceraldehyde-3-phosphate dehydrogenase* (*Gapdh*) levels. Library generation and bulk next-generation mRNA sequencing were performed by Psomagen, Inc. (Rockville, MD, USA) using TruSeq RNA Library Prep Kits (Illumina, San Diego, CA, USA) and the Illumina platform.

For the data analysis of the mRNA-seq data raw reads from fastq files were trimmed using TrimGalore, aligned to mm10 using Bowtie2 [95] and gene-level read counts were obtained using featureCounts [96]. Data were analyzed by Principal Component Analysis (PCA) using pcaExplorer [97]. DESeq2 [98] was used to identify differentially expressed genes (DEGs), which were defined as genes with a *p*-value of 0.05 and a >2-fold change comparing young to middle-aged or middle-aged to NLRP3 inhibition. Volcano Plots were generated using GGPlot2 (v3.4.0), Dplyr (v1.0.10) and Tidyverse (v1.3.2). Venn diagrams were generated using VennDiagram (v1.7.3), Dplyr (v1.0.10), Tidyverse v1.3.2.

Gene Set Enrichment Analysis (GSEA) was used to identify perturbed biological processes (GSE226796) (NCBI tracking system #23740500) [99]. Heatmaps were generated to demonstrate the differential expression of the genes part of individual gene sets (i.e., multiple pathways of the Hallmark data sets, the Cellular Senescence pathway of the C2.cp.reactome, and the Rodwell Aging Up/Down pathway of C2.cgp). The genes relevant to these pathways were extracted from the gene expression data used in the GSEA. The most upregulated genes in young, middle-aged, and MCC950-ionjected middle aged (NLRP3i) podocytes were mapped against one another. Heatmaps were generated using GGPlot2 (v3.4.0), Dplyr (v1.0.10), Tidyverse (v1.3.2), Patchwork (v1.1.2), ComplexHeatmap (v2.12.1). In all cases, the heatmaps were scaled between -1 and 1. All the R scripts are available upon request.

### Gene expression analysis of human kidneys

Kidney tissue was obtained from the unaffected parts of kidneys removed from patients undergoing surgery at the University of Michigan and processed immediately via the tissue procurement service of the Department of Pathology as described [19, 38]. Clinical data were obtained through the honest broker office of the University of Michigan as we have reported.^86^ Tissue was placed right away in RNAlater, micro-dissected into glomeruli and tubule-interstitial fractions, and isolated total RNA was used for gene expression analysis using polyA-RNA-sequencing as described [19]. Human kidney gene expression analysis studies were approved by the Institutional Review Board of the University of Michigan (IRB approval number: HUM00052918, HUM00165536). IRB 14-0019 from the University of Chicago was used for the immunostaining of human kidneys.

### Human kidney biopsies

Paraffin-embedded formalin fixed human kidney biopsies used were from the archives of Department of Pathology, University of Chicago (Chicago, IL, USA). These include healthy young human kidney tissue (23 years old), healthy middle age human kidney tissue (43, 45) years old and aged human kidney tissue (greater than 65) years old. These tissues underwent similar immunostaining protocol as were for mice.

### Human kidney organoids

Human kidney organoids derived from MANZ2 iPSCs were generated according to previously developed protocols [100–102]. Briefly, a cell culture treated 24 well plate (TPP 92012) was coated with anti-adherence solution (Fisher Scientific NC0488042) for 10 minutes, followed by 3 subsequent washes with sterile PBS. Day 14 organoids were placed into each well. Organoids were treated in three condition arms (*n* = 10–12 organoids/condition): untreated (control), 20 mg/mL cytotoxic antibody treated, or 20 mg/mL cytotoxic antibody + 10 mM MCC950 (MCE HY-12815A) treated every 24 hrs for a total of 48 hrs. Control treatment included equal volume of PBS to the antibody treatment arm. Organoids were harvested at day 16 by washing in PBS three times, removing supernatant, and resuspending in TRI reagent (Thermo Fischer AM9738). RNA was isolated using RNA isolation kit (Thermo Fisher 12-183-018A), and cDNA was synthesized using qScript cDNA SuperMix (Thermo Fisher NC0479275). qPCR was carried out and measured using QuantStudio 12K Flex and analysis was subsequently carried out in GraphPad Prism. Primer sequences for all qPCR markers were obtained from Massachusetts General Hospital PrimerBank: NRLP3-FOR, GATCTTCGCTGCGATCAACAG; NRLP3-REV, CGTGCATTATCTGAACCCCAC; IL1B-FOR, ATGATGGCTTATTACAGTGGCAA; IL1B-REV, GTCGGAGATTCGTAGCTGGA; CASP1-FOR, TTTCCGCAAGGTTCGATTTTCA; CASP1-REV, GGCATCTGCGCTCTACCATC; PYCARD-FOR, TGGATGCTCTGTACGGGAAG; PYCARD-REV, CCAGGCTGGTGTGAAACTGAA; CDKN2D-FOR, AGTCCAGTCCATGACGCAG; CDKN2D-REV, ATCAGGCACGTTGACATCAGC.

### Statistical analysis

Data are shown as the mean ± S.E.M. Student’s *t*-test was applied for comparisons between two groups. *P*-values < 0.05 were considered statistically significant. Multiple groups were compared using one-way ANOVA with post hoc Tukey HSD test. *P*-values < 0.05 represented statistically significant differences.

## Supplementary Materials

Supplementary Figures

Supplementary Table 1
