# Supplementary material for: Inhibiting NLRP3 signaling in aging podocytes improves their life- and health-span
Source: Aging (Albany NY). 2023 Jul 23;15(14):6658–89. doi: 10.18632/aging.204897 (PMC10415579; doi:10.18632/aging.204897)
Supplement: Supplementary Figures [file aging-15-204897-s001.pdf]

SUPPLEMENTARY Figures

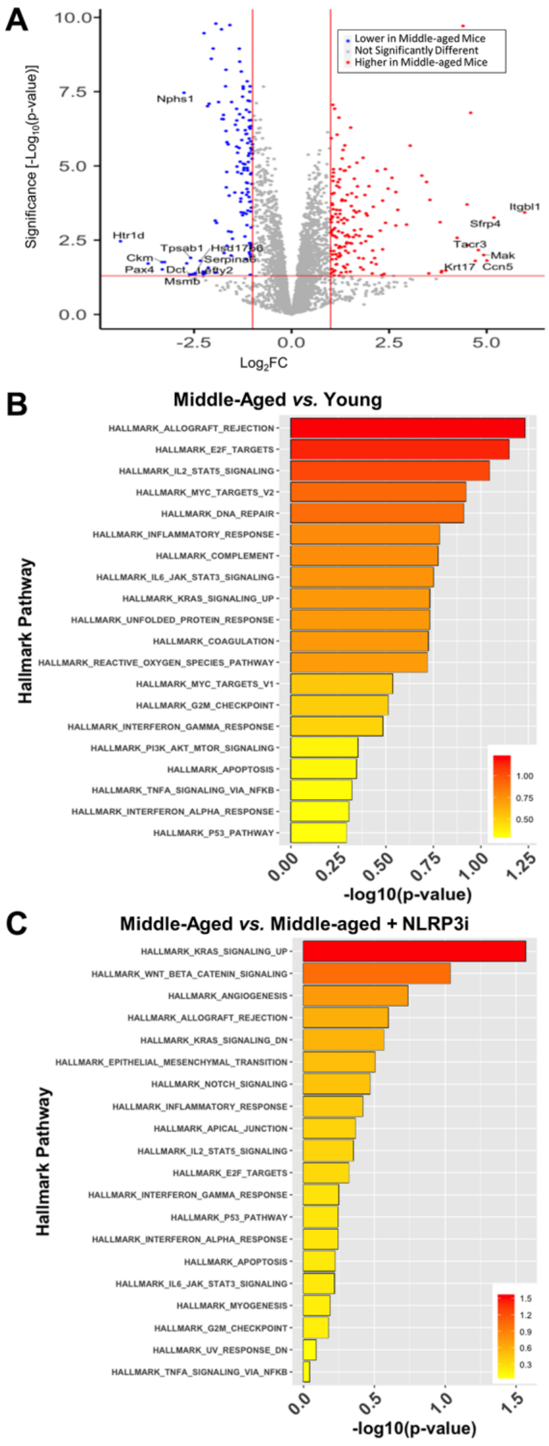

Supplementary Figure 1. Podocyte transcriptome analysis. (**A**) Volcano Plot comparing the young and the saline treated middle-aged podocyte transcriptomes; transcripts changed >2 and with a *p*-value > 0.05 are indicated in blue, when down-regulated in middle aged mice, and red, when up-regulated. (**B**, **C**) Bar graphs of the GSEA analysis of the Hallmark gene sets comparing young and saline-treated middle-aged podocytes (**B**) and saline- and MCC950 (NLRP3i)-treated middle aged podocytes.

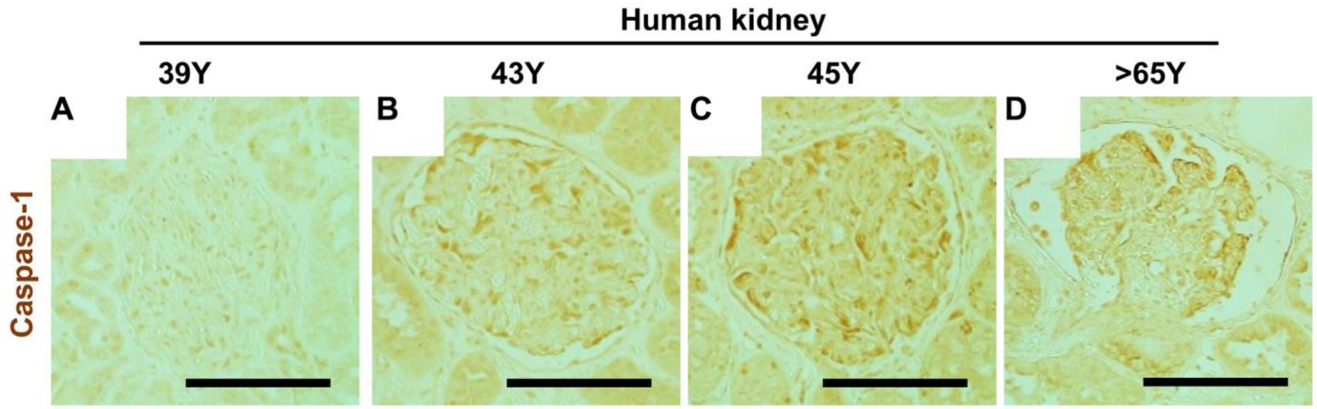

Supplementary Figure 2. Caspase-1 staining in healthy middle-aged human glomeruli. (**A**–**D**) Caspase-1 immunostaining (brown color) comparing kidneys of a 39, 43, 45 and more than 65 years old humans. Representative images are shown. Scale bars correspond to 25 μm.

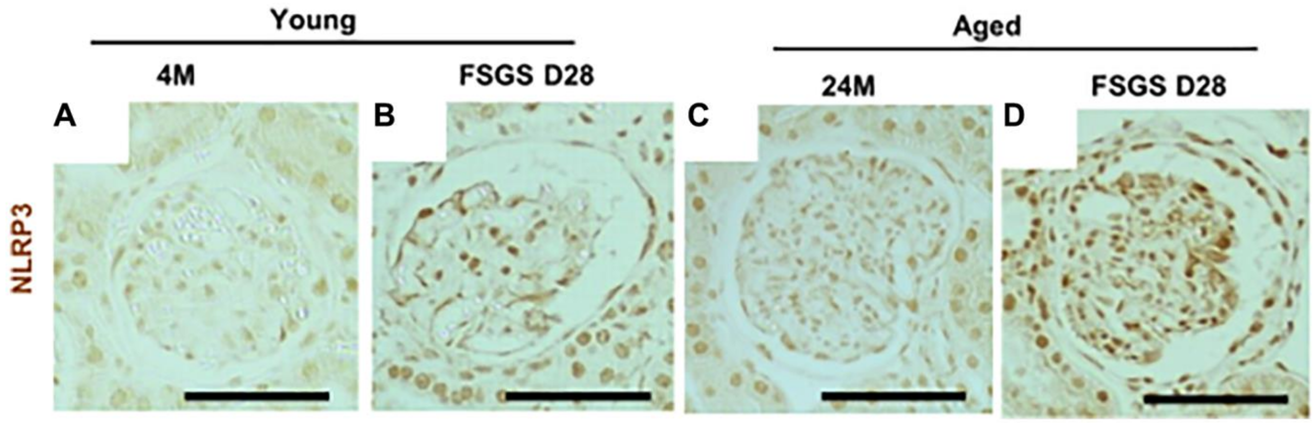

Supplementary Figure 3. NLRP3 staining in FSGS in young mice is augmented when disease is in aged mice. (**A**–**D**) Immunostaining for NLRP3 comparing glomeruli of control mice and mice with experimental FSGS at 28 days at young age (3 m) and aged (24 m). Note that no staining was detected in glomeruli of healthy young mice (**A**) but was increased in both podocytes and parietal epithelial cells in FSGS at young age (**B**); NLRP3 staining was elevated in glomeruli of healthy aged mice (**C**) and further augmented in those mice with experimental FSGS (**D**).

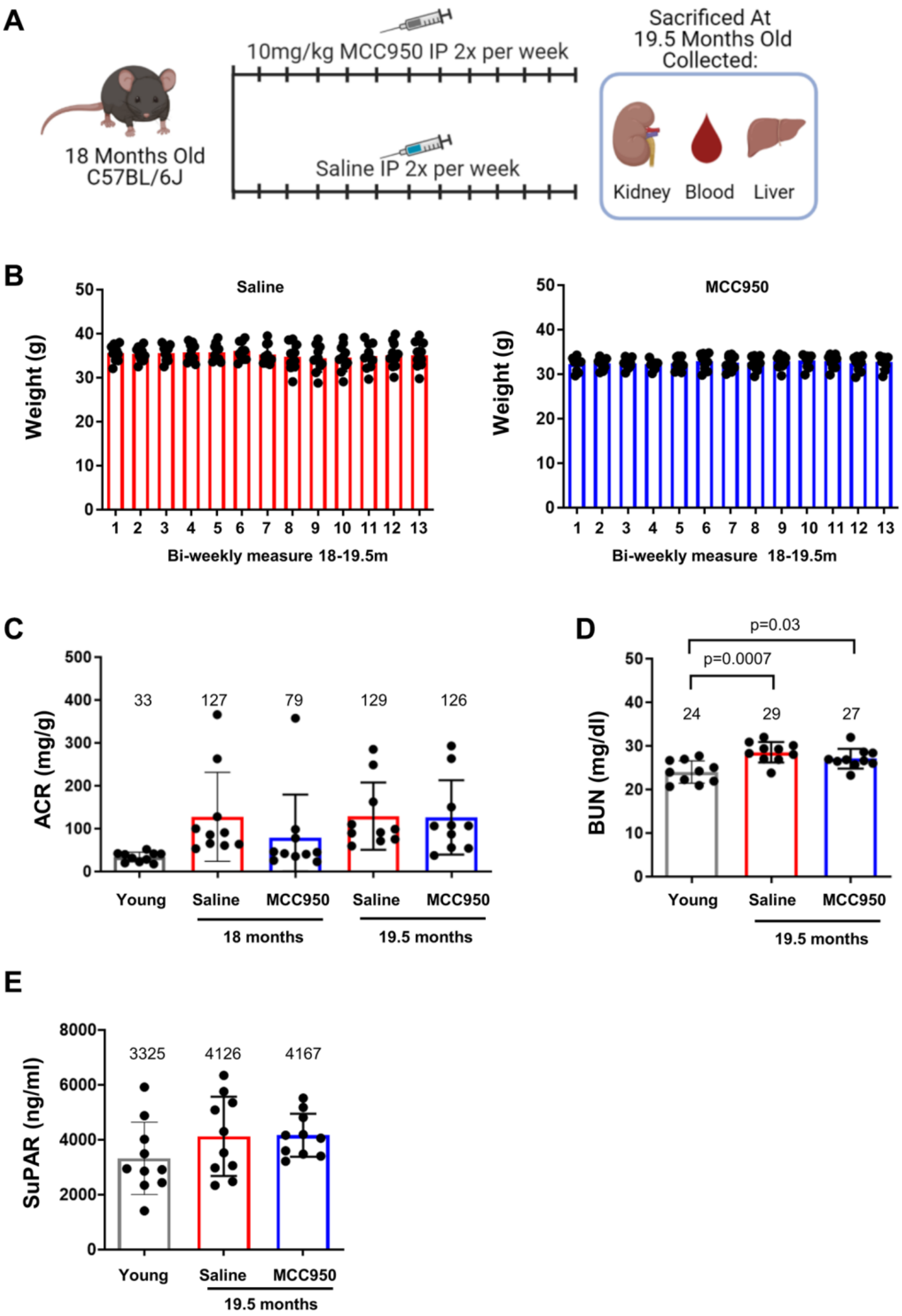

Supplementary Figure 4. Experimental design and clinical measurements. (**A**) Schematic of the treatment paradigm. Male 18 m C57BL/6J mice were divided into two groups to receive either MCC950 at 10 mg/kg (n = 10) or saline (n = 10), twice per week for 6 weeks, and sacrificed at 19.5 months of age and kidneys, blood and livers were collected for subsequent analyses. (**B**) Animals were weighed prior to each injection and at sacrifice and weight are depicted in the bar diagrams. Statistical analysis did not detect significant changes in weight in either the saline- or the MCC950-treatment for the duration of the experiment. (**C**) Bar diagram showing ACR levels throughout the experiment. Although there was a trend showing an increase in average albuminuria when comparing middle-aged to young mice, the difference is not significant by Student's *t*-test. No significant differences were observed between the saline and MCC950 treatment groups either at the 18 m pre-treatment and 19.5 m post treatment urine collection time points. (**D**) Bar diagram of the BUN levels comparing young to saline- or MCC950-treated 19.5 m middle-aged mice. Student's *t*-test demonstrated a significantly increased between young and both groups of middle-aged mice, but no statistical significant difference between the saline- and MCC950-treated mice. (**E**) Bar diagram of the SuPAR levels in young, and middle-aged saline- and MCC950-treated mice. No significant statistical difference was detected between any of the groups. In all graphs error bars are standard deviation and the mean levels are indicated by the number above the bars.

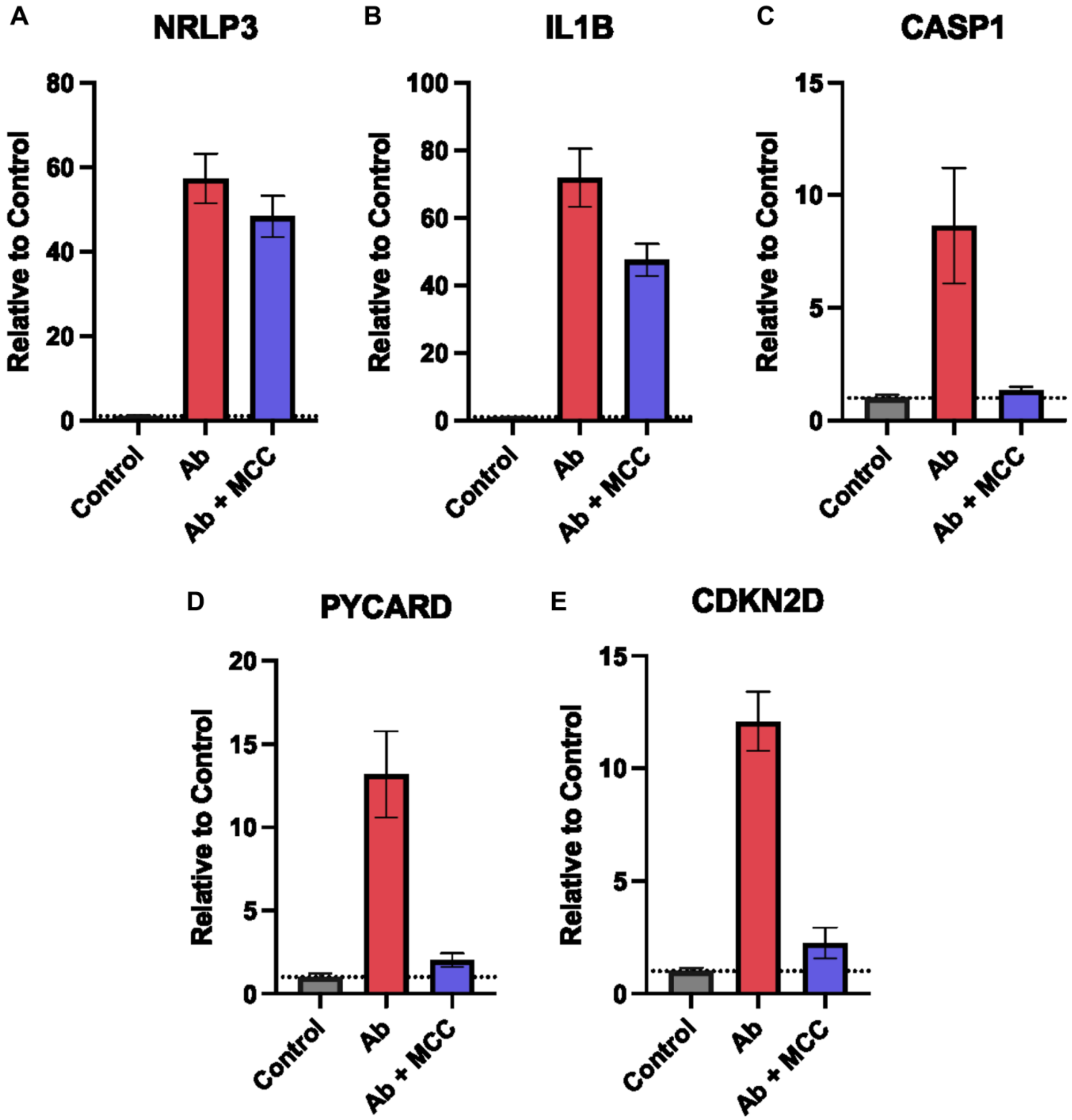

Supplementary Figure 5. NLRP3 signaling in injured human kidney organoids. Mature human kidney organoids at day 14 were treated with and without 20 mg/mL of cytopathic anti-podocyte antibody. Injured organoids were also treated with and without 10 mM MCC950. Organoids were analyzed by qPCR at day 16 and measured for expression of NRLP3 (**A**), IL-1β (**B**), CASP1 (**C**), PYCARD (**D**), and CDKN2D (**E**). The graphs represent 10–12 organoids and the error bar represents 3 technical replicas.

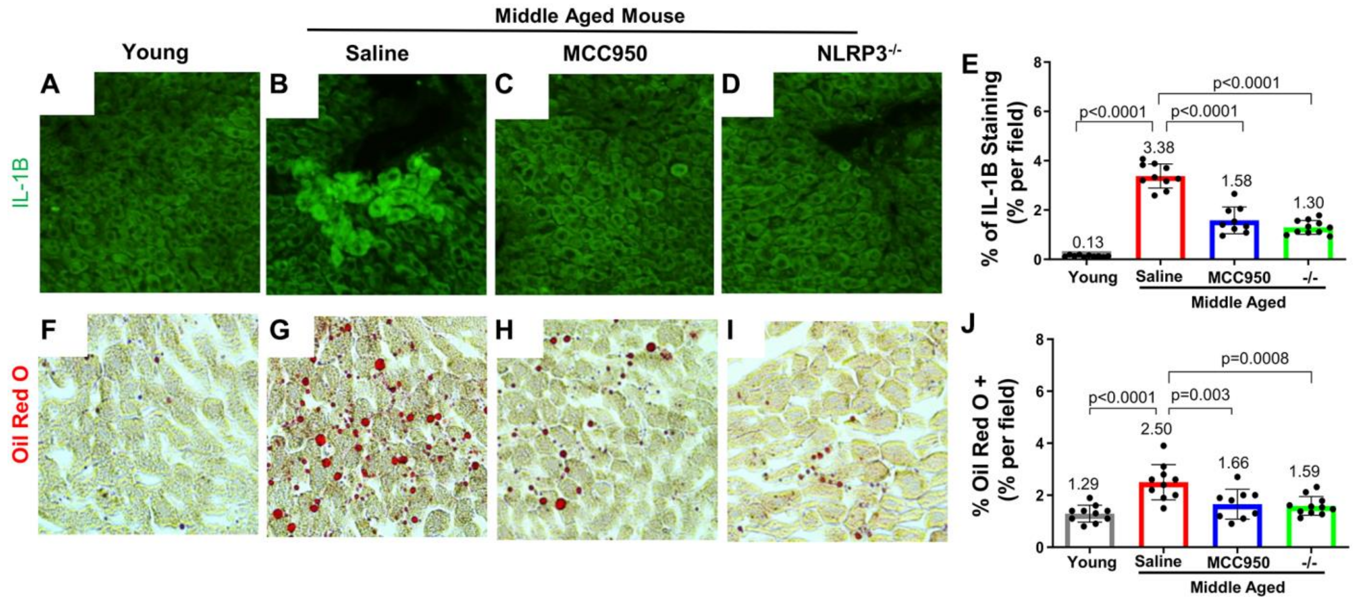

Supplementary Figure 6. Impact of MCC950 on the middle-aged mouse liver. (**A**–**E**) Livers of young, middle-aged saline- and MCC950-treated mice as well as age-matched Nlrp3 null (−/−) mice were compared by Interleukin-1 beta (IL-1β immunofluorescent (**A**–**D**, green), data were quantified as % of IL-1β-positive staining area per field (**E**) and oil red O staining (**F**–**I**, red) and quantified as the percent of oil red O positive area per field (**J**). Error bars are standard deviation, and the mean levels are stated by the number above the bars. Data were analyzed by pairwise comparisons using Student's *t*-test and the *p*-values are indicated above the bars. Note that both IL-1β and oil red O staining increased from young to middle-age and are reduced by either MCC950 treatment or NLRP3 ablation.
