# Supplementary material for: Inhibiting NLRP3 signaling in aging podocytes improves their life- and health-span
Source: Aging (Albany NY). 2023 Jul 23;15(14):6658–89. doi: 10.18632/aging.204897 (PMC10415579; doi:10.18632/aging.204897)
Supplement: Supplementary Table 1 [file aging-15-204897-s002.pdf]

SUPPLEMENTARY TABLE

Supplementary Table 1. List of antibodies.

Primary antibody	Antibody to identify	Raised in	Working dilution	Antigen retrieval buffer	Source/catalog number
**p57**	Podocytes	Rabbit	1:800	EDTA buffer pH6	Santa Cruz Biotechnology, Santa Cruz, CA, USA sc8298
**Collagen type IV**	Glomerular injury	Rabbit	1:200	EDTA buffer pH6	Southern Biotechnology, Birmingham, AL, USA 1430-01
**Synaptopodin**	Actin-associated protein in renal podocytes	Mouse	1:500	EDTA buffer pH8	Fitzgerald Industries International. Inc., Concord, MA RDI-PRO65194
**Nephrin**	Slit diaphragm of podocytes	Guinea pig	1:1500	EDTA buffer pH6	Fitzgerald Industries International. Inc., Concord, MA RDI-PROGPN2
**Podocin**	Podocytes	Rabbit	1:4000	EDTA buffer pH6	Abcam, Cambridge, MA, USA Ab50339
**WT1**	Podocytes	Rabbit	1:500	Citrate buffer pH6	Abclonal Science, Woburn, MA, USA A2446
**Nephrin (Y17-R)**	Extracellular Domain	Rabbit	1:100	MACS	MyBiosource.com MBA684100
**Nephrin (G17-H)**	Extracellular Domain	Rabbit	1:100	MACS	MyBiosource.com MBS684143
**Desmin**	Podocyte injury/stress marker	Rabbit	1:1000	Citrate buffer pH 6	Abcam, Cambridge, MA, USA ab15200
**VEGF-A**	Podocyte synthesis	Rabbit	1:200	Citrate buffer pH 6	Abcam, Cambridge, MA, USA ab52917
**NLRP3**	Formation of inflammasome	Rabbit	1:200	Citrate buffer pH6	Signalway Antibody, Greenbelt, Maryland, USA 49012-1
**Caspase-1**	NLRP3 downstream target	Rabbit	1:200	Citrate buffer pH 6	Enzo Life Science, Farmingdale, NY, USA ALX-210-804-C100
**IL-1B**	NLRP3 downstream target	Rabbit	1:200	Citrate buffer pH 6	Thermo Fisher Scientific, Waltham, MA, USA P420B
**IL-18**	NLRP3 downstream target	Rabbit	1:100	Citrate buffer pH 6	Rockland Immunochemicals, Gilbertsville, PA, USA 210-401-323
**GRP94**	Endoplasmic reticulum stress	Rabbit	1:200	Citrate buffer pH 6	Thermo Fisher Scientific, Waltham, MA, USA MA3-016
**Cleaved caspase 3 antibody**	Apoptosis	Rabbit	1:200	Citrate buffer pH 6	Cell Signaling, Danvers, MA, USA 9579S
**LC3**	Autophagy	Rabbit	1:200	Citrate buffer pH 6	Sigma-Aldrich, St. Louis, MO, USA L8918
**CD31**	Endothelial cells	Rat	1:200	Citrate buffer pH 6	Dianova, Hamburg, Germany DIA-310
**PV1**	Microvascular endothelial cells	Rabbit	1:100	EDTA buffer pH6	(PV1) antibody (1:100, BD Bioscience, San Jose, CA 550563

**α8 integrin**	Mesangial cells	Goat	1:100	Citrate buffer pH 6	R&D Systems Inc., Minneapolis, MN, USA BAF4076
**Lotus Tetragonolobus Lectin/LTL**	Proximal tubular epithelial cells	Tetragonolobus purpureus	1:500	Citrate buffer pH 6	Vector Labs, Burlingame, CA, USA B-1325-2
**Megalin/LRP2**	Proximal tubular epithelial cells	Rabbit	1:1000	Citrate buffer pH 6	Sino Biological, Wayne, PA, USA 106515-T08
**p16INK4a-N-terminal**	Senescence	Rabbit	1:1000	Citrate buffer pH 6	Abcam, Cambridge, MA, USA ab189034
**SA-β-galactosidase**	Senescence	Chemical component	1:1000	None	Cell Signaling, Danvers, MA, USA 9860S
**PAX8**	Parietal epithelial cells	Rabbit	1:500	EDTA buffer pH6	Protein Tech Group, Chicago, IL, USA 10336-1-AP
**CD44**	"Activated" parietal epithelial cells	Rat	1:50	Citrate buffer pH 6	BD Biosciences, San Jose, CA, USA 553131
**CD74**	"Activated" parietal epithelial cells	Rabbit	1:100	Citrate buffer pH 6	BD Biosciences, San Jose, CA), USA 555317
**pERK**	"Activated" parietal epithelial cells	Rabbit	1:100	Citrate buffer pH 7	Cell Signaling Technology, Beverly, MA, USA 9101S
**pS6RP**	To determine activation of the mammalian target of rapamycin (mTOR) pathway	Rabbit	1:100	Citrate buffer pH 6	Cell Signaling Technology, Beverly, MA, USA 2217S
**Phospho -STAT3**	Inflammatory Pathway	Rabbit	1:50	Citrate buffer pH6	Cell Signaling, Danvers, MA, USA 9145T
**Phospho-IKKα/β**	NF-κB Pathway	Rabbit	1:50	Citrate buffer pH6	Cell Signaling, Danvers, MA, USA 2697T
